# Refining shape and size of silver nanoparticles using ion irradiation for enhanced and homogeneous SERS activity

**DOI:** 10.1186/s11671-024-03994-x

**Published:** 2024-03-19

**Authors:** Laden Sherpa, Arun Nimmala, S. V. S. Nageswara Rao, S. A. Khan, Anand P. Pathak, Ajay Tripathi, Archana Tiwari

**Affiliations:** 1 Department of Physics, Sikkim University, Tadong, Gangtok, Sikkim 737102 India; 2 Centre for Advanced Studies in Electronics Science and Technology (CASEST), School of Physics, University of Hyderabad, Hyderabad, Telangana 500046 India; 3 Inter University Accelerator Centre, (IUAC), New Delhi, New Delhi 110067 India; 4 School of Physics University of Hyderabad, Hyderabad, Telangana 5000046 India; 5 Department of Physics, Institute of Science, Banaras Hindu University, Varanasi, Uttar Pradesh 221005 India

**Keywords:** Ion irradiation, Green synthesis, SERS, Enhancement factor

## Abstract

We present green synthesis of silver nanoparticles in water using unirradiated and Ag$^{15+}$ ion irradiated phytoextracts of *Bergenia Ciliata* leaf, *Eupatorium adenophorum* leaf, *Rhododendron arboreum* leaf and flower. The use of different plant extracts and their subsequent ion irradiation allow for successful refinement of nanoparticle size and morphology. Due to changes in reducing and capping agents the nanoparticle surface functionalization also varies which not only controls the morphology but also allows for surface oxidation and aggregation processes. In this work, we have synthesized silver nanoparticles which exhibit sizes in the range from 13 to 24 nm and having shapes like spherical, quasispherical, trigonal, hexagonal, cylindrical, dendritic assemblies, and porous nanoparticles. Owing to changes in the size and shape of the nanoparticles, their direct bandgap (2.05 eV - 2.48 eV) and local surface plasmon resonance (420 nm - 490 nm) could also be tuned. These nanoparticles are examined as SERS substrates, where their enhancement factors, limit of detection for methylene blue, and SERS substrate homogeneity have been tested. It has been observed the nanoparticles synthesized using unirradiated plant extracts present an enhancement factor of 10$^6$ with a limit of detection 10$^{-8}$ M. Whereas nanoparticles with refined morphology and shapes upon irradiation present high enhancement factors of >10$^7$ and detection limit down to 10$^{-9}$ M. In addition, uniformity in Raman spectra over the SERS substrates has been obtained for selected Ag NPs substrates synthesized using irradiated extracts with minimum relative standard deviation in enhancement factor < 12%.

**Supplementary Information:**

The online version contains supplementary material available at 10.1186/s11671-024-03994-x.

## Introduction

The size and shape of metal nanoparticles (NPs) play significant role in modifying and monitoring their electronic and optical properties. This allows optimized usage of NPs in designing surface-enhanced Raman scattering (SERS) sensors and other optical sensors which holds potential in environmental analysis, diagnostics, catalysis, etc. [1]. Numerous approaches have been employed to synthesize shape and size-controlled metallic nanoparticles such as silver, gold, and platinum. Several cost-effective methods for synthesis of silver nanoparticles (Ag NPs) have shown that the size, morphology, stability, and their chemical and physical properties have a strong dependence on the synthesis procedures and experimental conditions [2, 3]. The green synthesis approach emphasizes environmentally friendly practices, utilizing non-toxic chemicals, environmentally benign solvents, and renewable materials such as plant extracts which eliminates the limitations of NPs application [4]. The plant-derived biomolecules having functional groups such as amine, hydroxyl, carboxylic acids, thiol, and carbonyl groups, react with the metal ions to reduce it to nano-sized structures [5, 6]. All plant parts, including leaves, fruit, stems, bark, roots, and flowers have been used in the biosynthesis of various shapes and sizes of NPs which exhibits antioxidant, antimicrobial, bio-sensing, anticorrosive and catalytic characteristics [7–10]. In this report, we present a novel technique to modulate plant extract via swift heavy ion irradiation which in turn refines shape and size of NPs when synthesized from those extracts. Ag NPs have a distinct color as a function of their size, shape, and environment because of the changes in their localized surface plasmon resonance (LSPR) [11, 12]. The frequency and intensity of LSPR depend on the distribution of polarization charge across the nanostructure, which is essentially determined by its shape and size. As a result, metal nanostructure monitors the wavelengths of light it scatters and absorbs, which gets reflected in LSPR response [13, 14]. The synthesis of complex structures like rods, ribbons, sheets, dendrites, etc. arranged in a particular way and their growth mechanism have shown that such hierarchical materials possess improved optical properties originating from their building blocks [15, 16]. Such engineered nanostructures with anisotropic shapes and irregular surfaces present better Raman enhancement factors than conventional spherical NPs [17]. The morphology and size of engineered nanostructures are excellent for Raman scattering and increase their applicability as SERS sensors. Efficient SERS detection of analyte molecules has been observed for the morphologies like nanorods, triangular, hexagonal nanoplates, nanostars, etc. in which the enhancement factors (EF) of the order of 10$^5$ or more have been achieved [18]. Green synthesized nanoflowers, Ag dendrites synthesized using ion irradiated plant extracts and Ag-rGO nanocomposites, have presented enhancements of >10$^6$ [19–21]. The SERS sensitivity and enhancement are higher over cap-shaped Ag NPs, meso-flowers, and micro flowers and present EF >10$^7$ [22, 23]. Ultrasensitive substrates with EF >10$^9$ are obtained for DNA-mediated wire-like clusters, hybrid structures of GO/Ag NPs/PCu@Si, and Ag NP/GO [24–26].

In green synthesis, it is a challenge to precisely control the physical characteristics of the NPs as many plant-based metabolites are responsible for the reduction of metal ions and their stabilization. As a result, such techniques provide multifaceted particles having inhomogeneous shapes and sizes owing to which their physicochemical and optical characteristics are also heterogeneous [27]. Thus, a strategy is required for refinement of the reactants (phytomolecules in this case) that hold significant impact on the shape, size and efficacy of the NPs [28]. The phytoextracts responsible for reduction and capping of Ag ions undergo modifications upon irradiation via change in the intermolecular strength of functional groups. The changes in phytoextract is modulated by varying the irradiation fluence and hence the morphologies and sizes of resultant NPs can be tuned [19]. In addition, ion irradiation has also been employed to engineer nanoparticles alone by modifying their crystallinity, structure, and morphologies, and thereby tailoring their surface plasmon resonance [29].

In this work we present one-pot synthesis of Ag NPs by employing plant extracts in unirradiated and irradiated conditions using 200 MeV Ag 15$^{+}$ ion. Different ion fluences lead to modification in functional groups of the extract. The modified extracts are utilized to modulate the shape and size of Ag NPs. The irradiation of the extracts introduces anisotropy in size and morphology which are employed as SERS substrates for detection of methylene blue (MB). The sensitivity and homogeneity of the probe signal over the SERS substrate is guided by the anisotropy in shapes and sizes of the NPs. In this work an enhanced and uniform SERS activity is obtained when NPs are synthesized from irradiated extracts. The results presented in this work are either at par with or are better than other SERS substrates which are synthesized chemically [30, 31].

## Experimental details

The protocol for synthesis of Ag NPs was adapted from Sherpa et al., 2022 [19]. In summary, 1 mM of AgNO$_{3}$ were prepared in Millipore water. The plant samples, *Bergenia ciliata* leaf (BCL), *Eupatorium adenophorum* leaf (EL), *Rhododendron arboreum* leaf (RL) and flower (RF) were picked from Okhrey (27$^{\circ }$8’58"N, 88$^\circ$6’6"E), Sikkim, India. The plant samples were rinsed, dried and finely powdered. To prepare the plant extract, 1 g of powdered plant parts were taken in 10 mL of water in water bath at 90$^{\circ }C$ for 30 mins following which the extract was filtered. The filtered extracts were drop cast on silicon substrates and were subsequently irradiated with Ag$^{15+}$ ions of 200 MeV energy at IUAC, New Delhi. The thickness of thin film was $\sim$74(2) $\mu$m. Three different ion fluences of 5x10$^{11}$ ions/cm$^{2}$(I1), 1x10$^{12}$ ions/cm$^{2}$(I2) and 7x10$^{12}$ ions/cm$^{2}$(I3) were utilized for the irradiation of extracts. The irradiated extracts using I1, I2, and I3 ion fluences were labeled for BCL extracts as BCLI1, BCLI2, BCLI3, and so on. The irradiation details and sample codes are given in Table 1. For the synthesis of Ag NPs, 1 mL of the unirradiated extract was added to 10 mL of AgNO$_3$ solution and stirred for an hour. Similarly, for the synthesis of Ag NPs using the irradiated extracts, the thin films of irradiated extract were removed and dissolved in 1 mL of water which were later added to 10 mL of AgNO$_3$ solution. The NPs synthesized using the extracts irradiated with ion fluences I1, I2, and I3 were respectively labeled as AgI1 NPs, AgI2 NPs, AgI3 NPs, and the extracts used in the synthesis are given in the parenthesis. The synthesized Ag NPs were repeatedly centrifuged and purified using deionized water before further characterizations.

**Table 1:** Details of extracts, their irradiation, and the corresponding sample codes of Ag NPs synthesized using unirradiated and irradiated extracts

Irradiation condition	Fluence	Extract code	Synthesized
of extract			Ag NPs
Unirradiated	NA	BCL, EL, RL and RF	Ag NPs
Irradiated	5x10$^{11}$ions/cm$^{2}$	BCLI1, ELI1	AgI1 NPs
		RLI1, RFI1	
With Ag$^{15+}$200 MeV	1x10$^{12}$ions/cm$^{2}$	BCLI2, ELI2	AgI2 NPs
		RLI2, RFI2	
	7x10$^{12}$ions/cm$^{2}$	BCLI3, ELI3	AgI3 NPs
		RLI3, RFI3	

The morphology of the samples was determined using Transmission electron microscopy (TEM) (FET Technai G$^2$ T20). X-Ray diffraction (XRD) patterns were obtained by PANalytical XPert Pro diffractometer($\lambda$=1.54 Å ). UV–vis absorption, photoluminescence and FTIR spectra were recorded using PE $\lambda$750, LS55 and Spectrum 100 systems respectively.

Renishaw microRaman spectrometer was used to observed Raman spectra using continuous wave 785 nm laser. The samples were exposed to laser power of 3 mW under 50X objective for 10 s. For SERS investigation, dried Ag NPs were added to different batches of MB concentrations prepared in 10 mL of water and left for 24 h to ensure optimal adsorption on Ag NPs substrates. The mixture was then centrifuged and the precipitates were transferred onto a silicon substrate for SERS measurements. A normal Raman spectrum was also obtained on silicon substrate under similar experimental conditions using 1 mM MB.

## Results and discussion

### Effects of irradiation on particle size, morphology, and crystallinity

**Fig. 1:**
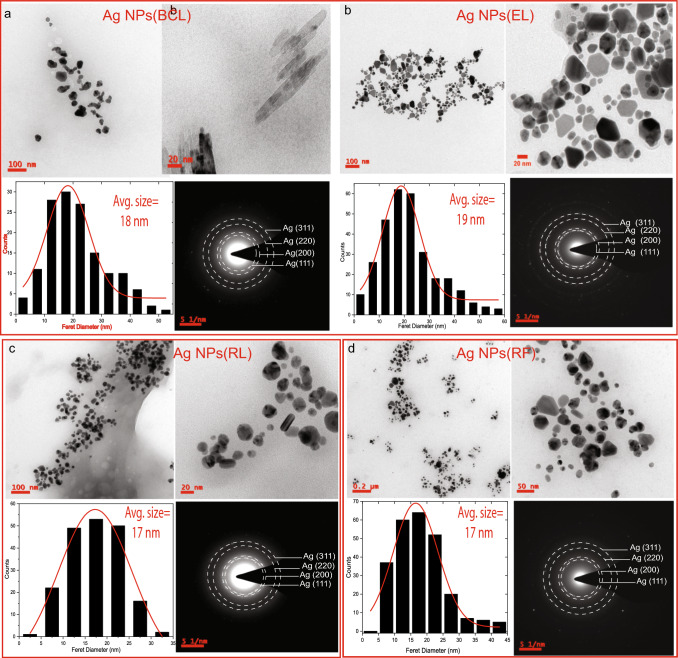
TEM micrographs showing particle morphology, size distribution histogram, and SAED pattern of **a** Ag NPs(BCL), **b** Ag NPs(EL) **c** Ag NPs(RL), **d** Ag NPs(RF)

TEM micrographs of Ag NPs(BCL) are shown in Fig. 1a. The particle morphology exhibits quasi-spherical, elongated, and unevenly shaped particles. The size distribution of Ag NPs(BCL) fitted with a Gaussian distribution shows that the average size of dispersed, non-agglomerated NPs is 18±1 nm. In addition, Ag NPs(BCL) also feature a sparse presence of elongated, rod-like nanostructures with an average diameter of 10±2 nm and having an average length of 71±4 nm. The observed morphology and its impact on the optical properties can be correlated in the later section where the nanorods introduced anisotropy in Ag NPs systems accounts for the presence of more than one LSPR (transverse and longitudinal) in the UV–vis absorption spectrum. SAED pattern of Ag NPs(BCL) shows the diffraction spots having interlayer spacing (*d*) of 0.23 nm, 0.20 nm, 0.14 nm, and 0.12 nm which are indexed as (111), (200), (220), and (311) reflections of Ag fcc phases [19, 32].

Fig. 1b shows TEM micrographs of Ag NPs(EL). The particle morphology exhibits spherically shaped and multifaceted NPs such as trigonal, hexagonal, and cylindrical particles. The average size of Ag NPs is found to be 19±1 nm. Ag NPs(EL) are crystalline as confirmed by SAED pattern where the observed diffraction spots correspond to (111), (200), (220), and (311) planes of fcc Ag. In Fig. 1c, TEM micrographs of Ag NPs(RL) show well-dispersed spherically shaped particles. In addition, sparse distribution of cylindrical NPs were also observed. The average size of Ag NPs(RL) is found to be 17±1 nm. Their SAED pattern reveals diffraction spots corresponding to (111), (200), (220), and (311) planes of fcc Ag phases. In Fig. 1d, TEM micrographs of Ag NPs(RF) reveal spherical, trigonal, cylindrical, and other irregular shapes. Similar to that of Ag NPs(RL), the average size of Ag NPs(RF) is 17±1 nm. The NPs crystallize in purely fcc Ag phases as confirmed by the SAED pattern. Apart from different sizes and morphology the TEM micrographs also show that the synthesized Ag NPs undergo agglomeration due to colloidal instability. The size-dependent instability leads to Ostwald ripening and coalescence of NPs [33]. Further, the anisotropic shapes exhibit different surface energies at different crystal facets, which also facilitate agglomeration in the dispersion medium (water) [34]. The details of size and morphology for all the synthesized Ag NPs are given in supplementary Table S1. In order to examine the effects of irradiated extracts on the Ag NPs, TEM micrographs of these NPs have also been examined.

**Fig. 2:**
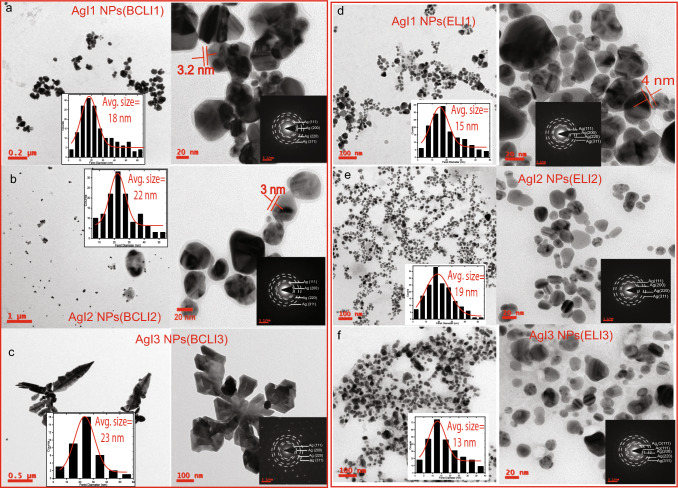
TEM micrographs showing particle morphology, size distribution histogram (inset), oxide thickness, and SAED pattern (inset) of **a** AgI1 NPs(BCLI1), **b** AgI2 NPs(BCLI2), **c** AgI3 NPs(BCLI3). TEM micrographs showing particle morphology, size distribution histogram (inset), oxide thickness/no oxidation, and SAED pattern (inset) of **d** AgI1 NPs(ELI1), **e** AgI2 NPs(ELI2), **f** AgI3 NPs(ELI3)

TEM micrograph of AgI1 NPs(BCLI1) is shown in Fig. 2a. Unlike Ag NPs(BCL) the NPs are mostly spherical and have a few hexagonal, cylindrical, and trigonal NPs. The size distribution histogram shows the average size of AgI1 NPs(BCLI1) is 18±1 nm. Similar to that of Ag NPs synthesized from unirradiated BCL extract the aggregation of NPs is also observed as they suffer colloidal instability [35]. A shell-like formation over the aggregated AgI1 NPs(BCLI1) surfaces is observed and ascribed to its surface oxidation [36, 37]. The thickness of the oxide layer is found to be $\sim$3.2 nm, as highlighted in the figure. TEM micrograph of AgI2 NPs(BCLI2) in Fig. 2b shows homogeneous and spherical particles of average size 22±1 nm. The agglomeration of AgI2 NPs(BCLI2) results in the formation of larger NPs, and undergo surface oxidation where the average shell thickness of the oxide layer is $\sim$3 nm. Figure 2c shows TEM micrograph of AgI3 NPs(BCLI3) where spherical NPs (sparsely present) and dendritic formation of NPs can be seen. The average size of spherical particles is found to be 23±1 nm. On the other hand, Ag dendrites feature sharp and rounded branches. The dendrites grow from a common core composed of many fused spherical NPs with an average diameter of 23 nm. The branch attachment outwards as the attachment grows faster at the edges following diffusion-limited aggregation. Some dendritic arms are selectively longer, revealing oriented attachment of NPs. Since different crystal faces of NPs have different surface energies, the adsorption energy along each face is non-identical. It leads to contrast in growth rates of dendritic arms in different directions [3, 15]. SAED pattern of AgI1 NPs(BCLI1), AgI2 NPs(BCLI2) and AgI3 NPs(BCLI3) are shown in Fig. 2a, b, c inset. Similar to that of Ag NPs(BCL) the diffraction spots have *d* spacing of 0.23 nm, 0.20 nm, 0.14 nm, and 0.12 nm which are indexed as (111), (200), (220), and (311) reflections revealing fcc Ag phases. Despite showing shell-like structures, the oxide phases were absent in SAED owing to the low yield of surface oxidized Ag NPs.

Figure 2d shows TEM micrograph of AgI1 NPs(ELI1). The dispersion of particles are mostly spherical, where some are elongated or trigonal in shape. The average size of these NPs is 15±1 nm. The aggregated AgI1 NPs(ELI1) show surface oxidation with an oxide layer encapsulating them in core-shell formation. The average shell thickness of the oxidized layer is $\sim$4 nm. In Fig. 2e, the morphology of AgI2 NPs(ELI2) have multiple shapes such as spherical, cylindrical, trigonal, etc. The size distribution histogram shows that the NPs are bigger than AgI1 NPs(ELI1), with an average size of 19±1 nm. Since the NPs are well dispersed, the number of NP aggregates is also lower, suggesting that the suspended NPs are well stabilized. TEM micrograph of AgI3 NPs(ELI3) in Fig. 2f shows well-dispersed particles with predominantly spherical shaped particles. The formation of a few irregularly shaped particles is attributed to NP agglomeration. The average size of AgI3 NPs(ELI3) is 13±1 nm. SAED pattern of AgI1 NPs(ELI1) and AgI2 NPs(ELI2) in Fig. 2d,e insets, correspond to the (111), (200), (220), and (311) planes of fcc Ag phases. SAED pattern of AgI3 NPs(ELI3) in Fig. 2f inset shows that in addition to Ag phases, (111) plane of Ag$_2$O phase is also present due to oxidation.

**Fig. 3:**
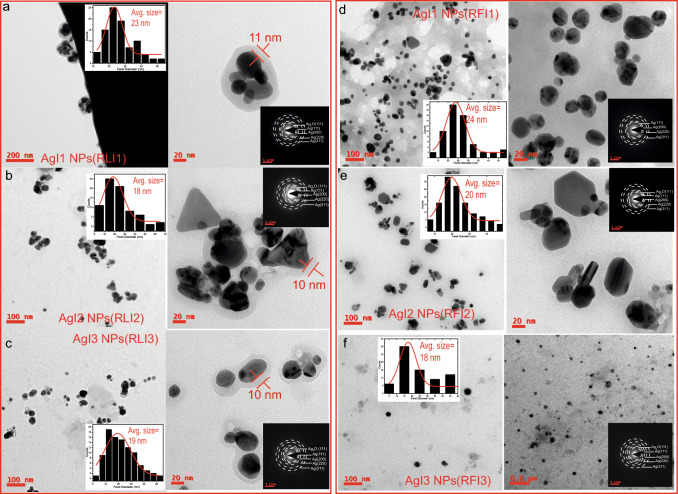
TEM micrographs showing particle morphology, size distribution histogram (inset), oxide thickness, and SAED pattern (inset) of **a** AgI1 NPs(RLI1), **b** AgI2 NPs(RLI2), **c** AgI3 NPs(RLI3). TEM micrographs showing particle morphology, size distribution histogram (inset), oxide thickness/no oxidation, and SAED pattern (inset) of **d** AgI1 NPs(RFI1), **e** AgI2 NPs(RFI2), **f** AgI3 NPs(RFI3)

In Fig. 3a, AgI1 NPs(RLI1) exhibit spherical, cylindrical, and elongated shapes of NPs. In AgI1 NPs(RLI1), aggregation and their clusters are observed in a core-shell-like formation. Individual NPs inside the shell have an average size of 23±1 nm. The Ag$_2$O shell over AgI1 NPs(RLI1) shields the Ag(core) from further oxidation [36]. The core-shell formation is in near-spherical shape. This near-spherical nature of the core-shell structure is attributed to the lattice mismatch between Ag and Ag$_2$O, which makes the surface amorphous and results in isotropic shell morphology [38, 39]. The average thickness of the shell is $\sim$11 nm and grows more significantly in size as the surface oxidized layer coalesces with neighboring shells. In Fig. 3b, TEM micrograph of AgI2 NPs(RLI2) shows spherical, trigonal, cylindrical, elongated, and irregularly shaped NPs. The average size of NPs is 18±1 nm. Similar to AgI1 NPs(RLI1), surface oxidation is observed in AgI2 NPs(RLI2) with an average shell thickness of $\sim$10 nm. AgI3 NPs(RLI3) have spherical and cylindrical shapes as shown in Fig. 3c. The average size of these particles is 19±1 nm. Similar to AgI1 NPs(RLI1), the aggregation of NPs is fully encapsulated by the oxide shells of thickness $\sim$10 nm. The SAED pattern of AgI1 NPs(RLI2), AgI2 NPs(RLI2), and AgI3 NPs(RLI3) are shown in Fig. 3a, b and c insets. Owing to the thick surface oxidized layer over Ag NPs the diffraction spots reveal the presence of both Ag and Ag$_2$O phases. Diffraction planes (111), (200), (220), (311) of Ag, and (111) plane of Ag$_2$O are observed in all the Ag NPs synthesized using RLI1, RLI2, and RLI3 extracts.

TEM micrograph of AgI1 NPs(RFI1) is shown in Fig. 3d, where AgI1 NPs(RFI1) are mostly spherical in shape. In addition, elongated, multifaceted particles and their aggregation are also observed. The average size of these particles is 24±1 nm. These Ag NPs are well dispersed with sparse aggregation and no visible surface oxidation over them. In Fig. 3e, TEM micrograph of AgI2 NPs(RFI2) exhibits hexagonal, cylindrical, and other faceted morphology in addition to spherical NPs of average size 20±1 nm. In AgI3 NPs(RFI3), TEM micrographs show well-dispersed, prominently spherical NPs in Fig. 3f with an average size of 18±1 nm. As observed in TEM micrographs, AgI3 NPs (RFI3) also show fragmented and porelike formation. The porosity in AgI3 NPs(RFI3) is introduced as a result of its oxidation. Owing to different rates of diffusion of reactants during oxidation reactions, pores are created due to the outward transport of fast-moving cations through the oxide layer and an inward balancing flow of vacancies in the vicinity of the metal-oxide interface. This is known as Kirkendall effect and is often seen in oxidized Ag NPs [40, 41]. SAED pattern of AgI1 NPs(RFI1) reveals Ag phases with planes along (111), (200), (220), and (311) directions. In AgI2 NPs(RFI2) and AgI3 NPs(RFI3) presence of Ag$_2$O phase in addition to Ag phases is observed (see Fig. 3e,f inset). The comparative details of size and morphology of all the synthesized Ag NPs using irradiated extracts are given in supplementary Table S1. The irradiation of plant extracts affects the morphology and size of the nanoparticles synthesized using them. It is observed that Ag NPs(BCL) have quasi-spherical and nanorod morphology. Upon irradiation, AgI1 NPs(BCLI1) and AgI2 NPs(BCLI2) exhibit spherical and polydisperse shapes. In addition, an oxide layer in core-shell formation of average thickness $\sim$3 nm is also observed. In AgI3 NPs(BCLI3), spherical particles and dendritic assembly are observed featuring sharp and rounded dendritic arms. The average size of the particles consistently increases from 18 to 23 nm from Ag NPs(BCL) to AgI3 NPs(BCLI3). Using EL extracts it is observed that Ag NPs(EL) exhibit spherical, trigonal, hexagonal, and cylindrical shapes while AgI1 NPs(ELI1) are mostly spherical and feature aggregates of multifaceted shapes, and an oxide layer of thickness 4 nm. AgI2 NPs(ELI2) maintain a similar morphology while the polydispersity in AgI3 NPs(ELI3) is reduced to spherical particles. The size of Ag NPs is observed to change from 19 to 13 nm from Ag NPs(EL) to AgI3 NPs(ELI3). Ag NPs synthesized from RL extracts present spherical, quasi-spherical, and cylindrical particles in Ag NPs(RL). AgI1 NPs(RLI1), AgI2 NPs(RLI2), and AgI3 NPs(RLI3) exhibit similar morphology and have an oxide layer of thickness $\sim$10 nm. The average size ranges from 17 to 23 nm from Ag NP(RL) to AgI3 NPs(RLI3). Synthesis of Ag NPs using unirradiated RF extracts show spherical shapes with sparse presence of spherical, quasispherical, trigonal, and cylindrical shaped Ag NPs(RF) and a similar morphology is observed in AgI1 NPs(RFI1). In contrast, AgI2 NPs(RFI2) exhibit multifaceted particles while AgI3 NPs(RFI3) present spherically porous NPs. Unlike other Ag NPs these NPs do not have an oxide layer over them irrespective of irradiation of the extracts. The average size ranges from 18 to 24 nm from Ag NP(RF) to AgI3 NPs(RFI3).

The morphology of Ag NPs synthesized from unirradiated and irradiated BCL exhibit anisotropic shapes, dendrites, and core-shell formation. The Ag NPs synthesized from EL extracts are multifaceted and have core-shell formation in some cases whereas those synthesized from uniradiated and irradiated RL extracts have prominent core-shell formation where the thickness of the oxidation shell is $\sim$10 nm. Further, in terms of homogeneity in size, it is observed that Ag NPs synthesized using unirradiated and irradiated RF extracts are well dispersed and homogeneous in size with standard deviation $\sigma$ $\sim$5 nm. Also, the morphology of these Ag NPs does not show a core-shell-like formation and exhibits predominantly spherical shapes in Ag NPs(RF), AgI1 NPs(RFI1), and AgI3 NPs(RFI3). Thus, for uniform size distribution and morphology, RF extracts present better control as reducing and capping agent than other plant extracts in both unirradiated and irradiated conditions. Here, we have shown that some extracts can be used for better control over size and morphology and further their refinement can be achieved through the irradiation of the extracts. Contrary to the preconceived notion that green synthesis yields no control over size and morphology, we have been able to improve uniformity in size and shape in AgI2 NPs(BCLI2), AgI3 NPs(ELI3), AgI1 NPs(RLI1), AgI1 NPs(RFI1) and AgI3 NPs(RFI3) through swift heavy ion irradiation.

**Fig. 4:**
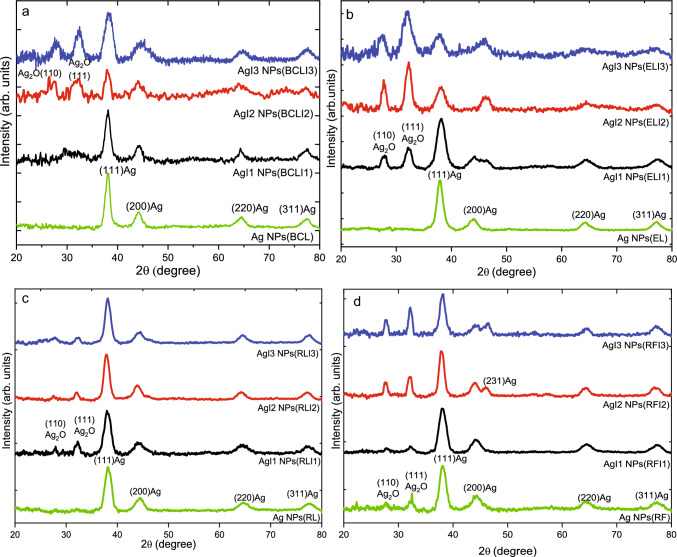
XRD patterns highlighting Ag and Ag$_{2}$O phases in Ag NPs, AgI1 NPs, AgI2 NPs and AgI3 NPs synthesized using unirradiated and I1, I2 and I3 fluence irradiated **a** BCL, **b** EL, **c** RL and **d** RF extracts

The crystallinity of the Ag NPs was further analyzed using XRD. XRD patterns of Ag NPs(BCL) are shown in Fig. 4a. It exhibits diffraction peaks at 38.1$^{\circ }$, 44.1$^{\circ }$, 64.4$^{\circ }$ and 77.4$^{\circ }$ where the maximum intensity peak is observed at 38.1$^{\circ }$. The *d* spacings obtained from the diffraction peaks are 2,36Å, 2.05Å, 1.45Å, and 1.236Å corresponding to (111), (200), (220), (311) planes of fcc Ag crystal [32]. AgI1 NPs(BCLI1) show similar diffraction peaks at 38.1$^\circ$, 44.2$^\circ$, 64.4$^\circ$ and 77.3$^\circ$ which correspond to the (111), (200), (220), and (311) planes of the fcc Ag crystal. In AgI2 NPs(BCLI2) apart from Ag phases, peaks at 27.2$^\circ$ and 32.0$^\circ$ correspond to (110) and (111) planes of cubic Ag$_2$O phase. Similarly, both Ag and Ag$_2$O phases are observed in AgI3 NPs(BCLI3). The intense diffraction peak is observed along (111) plane of fcc Ag phase in all the synthesized Ag NPs. Using Debye-Scherrer equation and the peak broadening in both Ag$_2$O and Ag phases, the crystallite size of Ag NPs was evaluated and is given in Table S2. The crystallite size in Ag NPs(BCL) is found to be 6.17 nm whereas in AgI1 NPs(BCLI1) it is found to be 6.9 nm. From AgI1 NPs(BCLI1) to AgI3 NPs(BCLI3), the crystallite size associated with Ag phases decreases. Because of surface oxidation, the crystallite size of Ag$_2$O phases increases from AgI2 NPs(BCLI2) to AgI3 NPs(BCLI3).

Similar to Ag NPs(BCL), XRD patterns of Ag NPs(EL) and Ag NPs(RL) in Fig. 4b, c show only fcc Ag phases. However, upon irradiation, both Ag$_2$O and Ag phases were observed in all the samples. The intense diffraction peak shifts from (111) plane of Ag to (111) plane of Ag$_2$O phase in AgI2 NPs(ELI2) and AgI3 NPs(ELI3) revealing increased oxidation upon using irradiated ELI2 and ELI3 as reducing agents. The calculated crystallite size for both Ag and Ag$_2$O phases reveal crystallite size of Ag phases decreases from AgI1 NPs(ELI1) to AgI3 NPs(ELI3) while similar crystallite size of Ag$_2$O is observed in them. Unlike Ag NPs synthesized from irradiated BCL and EL extracts, the peak intensity of Ag$_2$O phases remains constant in AgI1 NPs(RLI1), AgI2 NPs(RLI2), and AgI3 NPs(RLI3) and the intense peak remains along (111) plane of Ag phase. In Fig. 4d, XRD patterns reveal the presence of both Ag$_2$O and Ag phases in Ag NPs synthesized from unirradiated as well as irradiated RF extracts. An additional peak is observed at $\sim$46.1$^\circ$ in AgI2 NPs(RFI2) and AgI3 NPs(RFI3) which corresponds to (231) plane of fcc Ag phase [42, 43]. The intensity of diffraction peaks corresponding to Ag$_2$O phases increases from AgI1 NPs(RFI1) to AgI3 NPs(RFI3), due to increase in the extent of oxidation.

From Ag NPs to AgI3 NPs, the broadening of diffraction peaks and minute shifts are observed in multiple XRD peaks. It is associated with the confinement of NPs in the nano-regime and the lattice strain/stress imposed by the agglomeration, oxidation, and self-assemblies of the NPs as confirmed by TEM [44, 45]. The details of peak positions and crystallite size of Ag$_2$O and Ag phases in these Ag NPs are given in supplementary Table S2. The impact of size, morphology, crystallinity, and oxidation on the optical properties of the Ag NPs are investigated using UV–vis absorption and PL spectroscopy.

## The effects of irradiation on optical properties

**Fig. 5:**
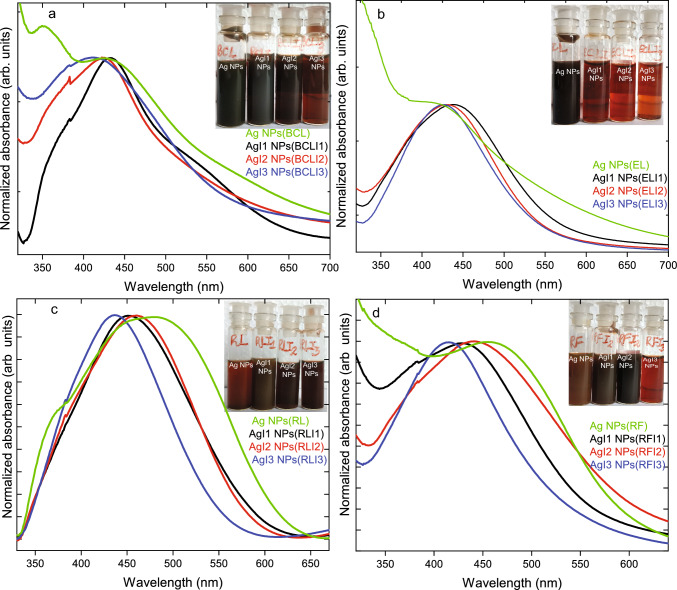
UV–Vis absorption spectra of Ag NPs synthesized from unirradiated, I1, I2, I3 fluence irradiated **a** BCL, **b** EL, **c** RL and **d** RF extracts. The absorption spectra of Ag NPs, AgI1 NPs, AgI2 NPs, and AgI3 NPs are color-coded as green, black, red, and blue respectively. Figure inset shows color of colloidal solution in sequential order as observed in Ag NPs, AgI1 NPs, AgI2 NPs and AgI3 NPs

UV–vis absorption spectrum of Ag NPs(BCL) is shown in Fig. 5a which presents absorption between 300-600 nm. Upon deconvolution, peaks are observed at 356±1 nm, 424±4 nm, 460±4 nm having FWHM 37±1 nm, 48±1 nm, and 65±1 nm respectively (see supplementary Fig.S1). The absorption corresponds to the characteristic local surface plasmon resonance (LSPR) due to the coupled vibration of free electrons on metal Ag NPs surface which resonates with the electromagnetic waves [46]. The two distinct LSPR bands in Ag NPs(BCL) indicate the presence of longitudinal and transverse plasmons [47]. This is in line with TEM micrographs where spherical, irregularly shaped particles and nanorod-like structures are observed. Upon synthesis, the color observed in the Ag NPs colloidal solution is shown in the inset. Ag NPs(BCL) colloidal solution exhibits a dark greenish-brown color. The color observed in Ag NPs colloidal solution depends on the presence of different morphologies and sizes and surface functionalization of the NPs [48]. Upon irradiation, AgI1 NPs(BCLI1), AgI2 NPs(BCLI2), and AgI3 NPs(BCLI3) subsequently exhibit light greenish-brown color, dark brown color, and reddish-brown color. Ag NPs synthesized from irradiated BCL extracts exhibit strong LSPR in aqueous solution in region 350–700 nm. In AgI1 NPs(BCLI1), the intense absorption peak is observed at 423±1 nm with FWHM 86±2 nm (upon deconvolution). Similarly, a narrow absorption peak at 423±1 nm having FWHM 48±1 nm is observed in AgI2 NPs(BCLI2). In AgI3 NPs(BCLI3), intense absorption is centered at 457±3 nm with FWHM 106±3 nm. The broad LSPR and its extension towards the longer wavelength are associated with large, multifaceted particles and wide distribution of NP sizes as they contribute to LSPR arising from various dipolar and quadrupolar resonances [49, 50].

In Fig. 5b, absorption spectra of Ag NPs synthesized from unirradiated and irradiated EL extract show broad absorption in the region 350–600 nm. The figure inset shows the color observed in the colloidal solution upon the synthesis of Ag NPs. A slight variation of color from dark brown in Ag NPs(EL), brown in AgI1 NPs(ELI1), reddish-brown in AgI2 NPs(ELI2), and light brown in AgI3 NPs(ELI3) colloidal solutions are observed. The broad LSPR and color variation is essentially due to anisotropy in shape, size, and aggregation of NPs [51]. These intense LSPR peaks are centered at 448±1 nm, 427±2 nm, 459±19 nm and 420±1 nm in Ag NPs(EL), AgI1 NPs(ELI1), AgI2 NPs(ELI2), and AgI3 NPs(ELI3) respectively. In Fig. 5c, Ag NPs synthesized from unirradiated and irradiated RL extract exhibit a broad absorption between 350-650 nm. The color of the colloidal solution shows a reddish-brown color in Ag NPs(RL) followed by muddy brown color in AgI1 NPs(RLI1) and dark brown color with a faint variation in AgI2 NPs(RLI2) and AgI3 NPs(RLI3). Owing to various shapes and sizes of Ag NPs the deconvolution of absorption peaks reveal intense peaks at 497±1 nm, 486$\pm 12$ nm, 480±11 nm, and 467±13 nm in Ag NPs(RL), AgI1 NPs(RLI1), AgI2 NPs(RLI2), and AgI3 NPs(RLI3) respectively. Similarly, in Fig. 5d absorption at 350–600 nm is observed for Ag NPs(RF), AgI1 NPs(RFI1), AgI2 NPs(RFI2), and AgI3 NPs(RFI3) and exhibit different color of colloidal solution such as brown color in Ag NPs(RF), a muddy brown color in AgI1 NPs(RFI1), dark brown color in AgI2 NPs(RFI2), and light brown in AgI3 NPs(RFI3) as shown in figure inset due to divergent shapes and sizes present in these NPs. The broadening of LSPR peaks results from the non-uniform shape and size distribution of synthesized Ag NPs. The effect of irradiation causes the plant phytomolecules to change. As these biomolecules are utilized to reduce Ag NPs, their chemical modification alters the synthesis process, leading to change in color of their colloidal solution and LSPR.

**Fig. 6:**
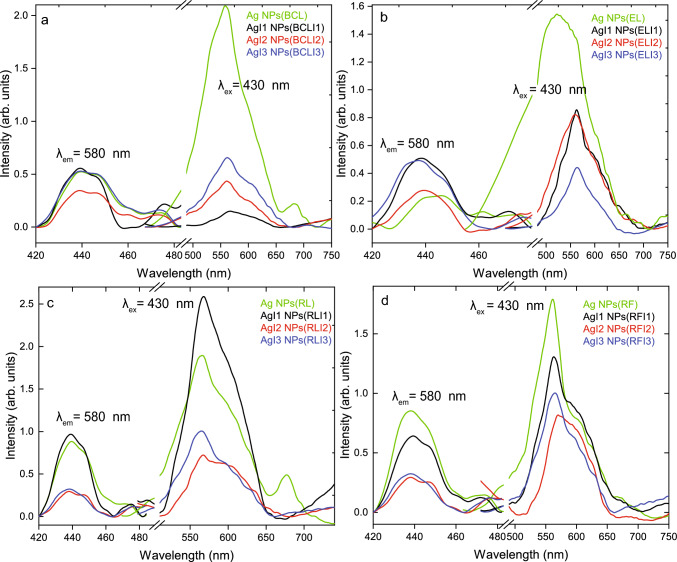
PL and PLE spectra of Ag NPs (green), AgI1 NPs (black), AgI2 NPs (red), and AgI3 NPs (blue) synthesized using unirradiated and I1, I2, I3 fluence irradiated **a** BCL, **b** EL, **c** RL and **d** RF extracts

For all synthesized Ag NPs, PL was monitored using an excitation wavelength($\lambda _{ex.}$) 430 nm and PLE using emission wavelength($\lambda _{em.}$) 580 nm. PL of Ag NPs(BCL), AgI1 NPs(BCLI1), AgI2 NPs(BCLI2) and AgI3 NPs(BCLI3) are shown in Fig. 6a. A broad emission is observed in the visible region 450–700 nm. The broad PL emission accounts for the heterogeneity in the size dispersion of NPs. Upon deconvolution, the most intense peaks are observed at 542±1 nm, 564±1 nm, 563±1 nm, and 559±1 nm in Ag NPs(BCL), AgI1 NPs(BCLI1), AgI2 NPs(BCLI2) and AgI3 NPs(BCLI3) respectively (see supplementary Fig. S3,S4). The variation in intensity depends on particle size, surface atoms, oxidation, and polarity of the ligand [52]. The weak PL exhibited by these Ag NPs are related to interband transitions where Fermi level electrons radiatively recombine with *sp* or *d* band holes rather than intraband transitions which occur for quantum confinement of Ag NPs systems [50, 53]. In bulk metal, the conduction and valence bands overlap, forming a continuous spectrum. In metal NPs, the interband transitions are more prominent than their bulk counterparts as the number of atoms is drastically reduced and is responsible for PL in Ag NPs [54, 55]. Broad PLE peaks centered around $\sim$448±1 nm are observed in all samples. The region of PLE is similar to that of LSPR exhibited by Ag NPs. Similarly, PL and PLE of Ag NPs synthesized using unirradiated and irradiated EL, RL, and RF extracts are shown in Fig. 6b, c, d. PL peaks with varying intensities are centered around $\sim$550 nm. PL of Ag NPs(EL) is comparatively broader than that of other Ag NPs. This can be correlated to the presence of anisotropically shaped multifaceted particles as observed from TEM micrographs. The broad PLE in these Ag NPs is also centered at $\sim$440 nm. Like PL, the variation in PLE intensities is observed in these samples. This is due to variation in the population of electrons from *d* to *sp* band during excitation.

The estimated optical band edges from Tauc plot are shown in supplementary Fig.S6 which reveals that band-gap in Ag NPs(BCL), Ag NPs(EL), Ag NPs(RL), and Ag NPs(RF) range from 2.05$-$2.19 eV. For those NPs synthesized from irradiated BCL, EL, RL, and RF extracts, the band gap appears to increase and ranges from 2.19$-$2.29 eV, 2.32$-$2.42 eV, 2.22$-$2.35 eV, and 2.19$-$2.48 eV, respectively. The obtained optical band gaps in the synthesized Ag NPs are in line with those reported previously in the literature [32, 56, 57]. The schematic in Fig.S7 reveals that the luminescence in these Ag NPs is associated with defect-related states. The visible luminescence includes transitions from states below the band edge revealing the presence of defect states in these Ag NPs. The localized defect states in Ag NPs act as radiative centers for below band-gap transitions as verified by multiple reports [19]. Such emissions occur due to the presence of intrinsic defects that have been introduced due to Ag NPs oxidation and their agglomeration/aggregation [58, 59].

### Effects of irradiation on functional group analysis

The Ag NPs exhibit different morphologies and sizes based on the functionalization provided by the phytomolecules. Upon irradiation, these functionalization changes, and hence the size and shape of Ag NPs also change. The phytoextracts play a significant role in the reduction, capping, and surface functionalization of Ag NPs, which are studied using FTIR.

**Fig. 7:**
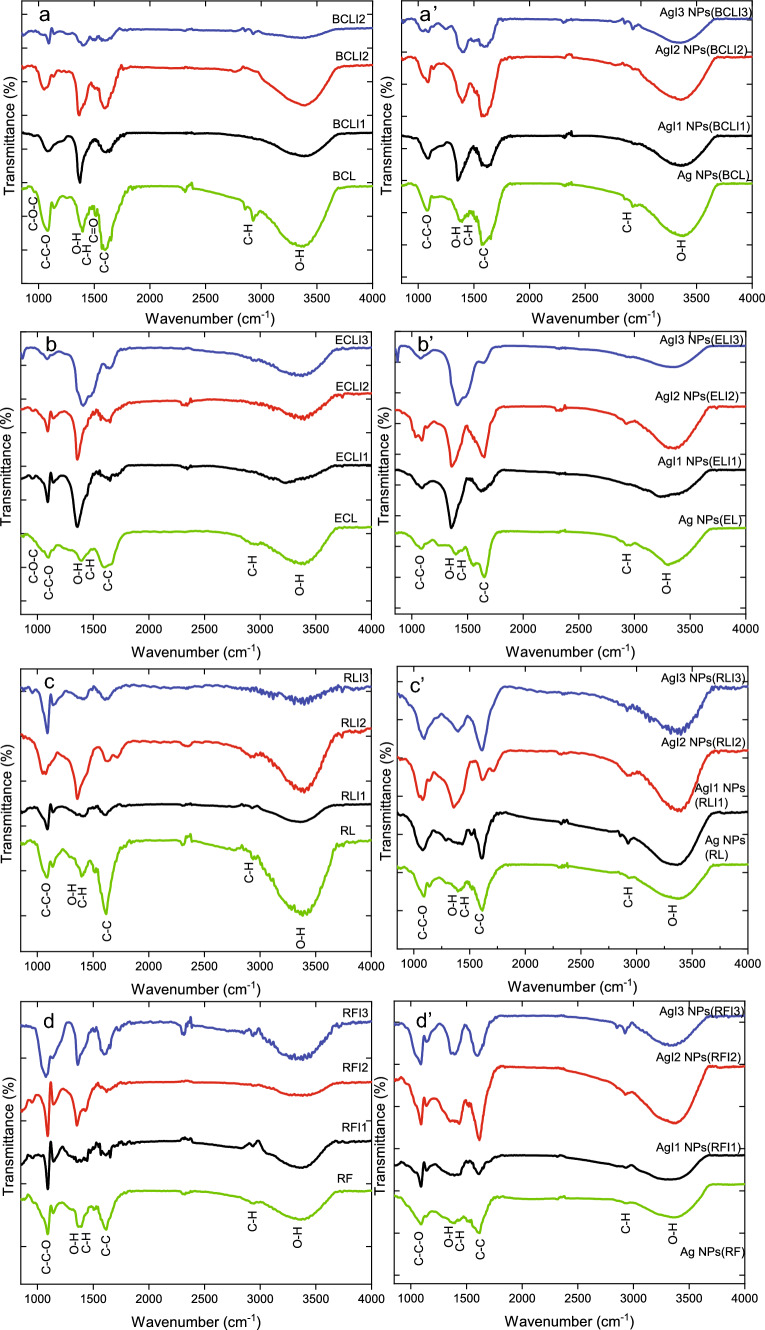
FTIR spectra of unirradiated and I1, I2, I3 fluence irradiated **a** BCL, **b** EL **c** RL, **d** RF extracts. FTIR spectra of Ag NPs, AgI1 NPs, AgI2 NPs, and AgI3 NPs synthesized using unirradiated and irradiated a’. BCL, b’. EL c’. RL, d’. RF extracts

The chemical composition of plant extracts and the involvement of different phytomolecules responsible for the reduction and capping is examined here. Owing to the presence of phytoextracts such as phenol, alcohol, terpenoids, fatty acids, carboxylic acids, and flavonoids, various functional groups have been identified in plant extracts. In Fig. 7a, FTIR spectra of unirradiated and irradiated BCL extract are shown. In BCL extract FTIR peaks are observed at 959 cm$^{-1}$, 1082 cm$^{-1}$, 1141 cm$^{-1}$, 1252 cm$^{-1}$, 1399 cm$^{-1}$, 1442 cm$^{-1}$, 1513 cm$^{-1}$, 1597 cm$^{-1}$, 2859 cm$^{-1}$, 2935 cm$^{-1}$ and 3362 cm$^{-1}$. The peak at 959 cm$^{-1}$ is ascribed to asymmetric stretching vibrations of C–O–C bond. The branched peaks at 1082 cm$^{-1}$, 1141 cm$^{-1}$, and 1252 cm$^{-1}$ correspond to C–C–O stretch owing to the presence of phenols. The split peaks at 1399 cm$^{-1}$ and 1442 cm$^{-1}$ correspond to coupled O-H and C–H vibrations as observed in alcohols. The peak at 1597 cm$^{-1}$ corresponds to C–C aromatic stretch whereas sharp peaks at 2859 cm$^{-1}$ and 2935 cm$^{-1}$ correspond to C–H vibrations in aromatics and alkanes. The peak corresponding to O–H groups is observed at 3362 cm$^{-1}$ [60, 61]. The obtained FTIR peaks and assigned functional groups are given in supplementary Table S3. In comparison to the unirradiated BCL extract, functional groups such as C–O–C, C–C–O, C–C, C–H, and O–H groups in BCLI1, BCLI2 and BCLI3 extract are reduced in intensity. The peak corresponding to coupled O–H and C–H peak are observed as narrow and intense in BCLI1 and BCLI2 extracts. As the phytomolecules are modified upon irradiation, the functional groups such as C–C–O, coupled O–H and C–H, C–C and O–H are severely modified. The phytomolecules present in both unirradiated and irradiated extracts help in reduction of Ag$^+$ ions into Ag$^0$ NPs and also act as their capping agent against the agglomeration [62]. Since the functional groups are modified due to irradiation, their utilization in the reduction and capping may result in synthesis of differently sized and shaped Ag NPs, as discussed in the following paragraph.

In Fig. 7a’, FTIR spectra of Ag NPs(BCL), AgI1 NPs(BCLI1), AgI2 NPs(BCLI2), and AgI3 NPs(BCLI3) are shown. Like those of the extracts, similar peaks are observed in Ag NPs. The observed variation in peaks corresponding to different functional groups is ascribed to their role in the reduction and/or capping of Ag NPs. Significant shifts in peak position are observed for coupled O–H, C–H, C–C, C–H, and O–H peaks. C–C–O and coupled O–H, C–H, and C–H peaks are reduced in intensity for Ag NPs(BCL). Further, the participation of C–O–C group in the reduction of Ag ions is suggested by its absence in Ag NPs(BCL), AgI1 NPs(BCLI1), AgI2 NPs(BCLI2) and AgI3 NPs(BCLI3). C–C–O peak becomes narrow in AgI1 NPs(BCLI1), AgI2 NPs(BCLI2) and broad in AgI3 NPs(BCLI3). In AgI1 NPs(BCLI1) coupled O–H and C–H peaks become broad, peak intensity of C–C and O–H peaks are enhanced and O–H peak is notably shifted. In AgI2 NPs(BCLI2), C–C peak is broad, the coupled O–H and C–H peaks are reduced in intensity, and a shift is observed for both C–H and O–H peaks. In AgI3 NPs(BCLI3), most of the peaks are narrow and enhanced in intensity. The observed changes in peaks are due to the interaction of available functional groups with the Ag ions. Owing to these interactions, the chemical environment and the intermolecular strength of the functional groups are affected [63]. The suppression of FTIR peaks corresponding to the functional groups in the Ag NPs is due to binding with Ag ions and their reduction into Ag$^0$. Shifts in peak positions are observed for the majority of functional groups such as C–C–O, coupled O–H and C–H, C–C, C–H, and O–H peaks. However, C–H and O–H peaks show notable shifts. The increase in peak intensities is related to the increase in the number of functional groups associated with the Ag NPs via surface functionalization. Meanwhile, the least shifted and unmodified functional groups in the FTIR spectra indicate that they may be surface-bound or may have been utilized in stabilizing the Ag NPs [64]. A schematic of utilizing the polyphenolic compounds such as epicatechin (a phenol) and quercetin (a flavonoid) present in unirradiated BCL extract in the reduction of Ag ion and synthesis of NPs is shown in supplementary Fig.S8.

Similarly, FTIR spectra of unirradiated and irradiated EL, RL, and RF extracts and the synthesized Ag NPs are shown in Fig. 7b, b’,c, c’,d, d’. The obtained FTIR peaks and the assigned functional groups have been reported in Tables S4, S5, S6. It is observed that the reduction and/or capping of Ag NPs(EL) is mediated by functional groups such as C–O–C, C–C, C–H, and O–H groups. Upon synthesis of Ag NPs, the C-O-C peak is absent in Ag NPs(EL), AgI1 NPs(ELI1), AgI2 NPs(ELI2), and AgI3 NPs(ELI3). The branched C–C–O peak is broadened in AgI1 NPs(ELI1) and AgI2 NPs(ELI2). Peak intensity of C–C, C–H, and O–H is enhanced in AgI2 NPs(ELI2). C–C peak broadens while the O–H peak narrows with a notable shift in O–H peak in Ag NPs synthesized using unirradiated and irradiated EL extracts.

Similarly, with respect to the extract, shifts in peak positions and changes in peak shapes are observed for C–C–O, O–H, C–H, coupled O–H, C–H, and C–C peaks in Ag NPs synthesized using unirradiated and irradiated RL extracts. In Ag NPs(RL), all the observed FTIR peaks are broadened and reduced in intensity. An increase in intensity is observed only for C–C, C–H, and O–H peaks in AgI1 NPs(RLI1) and AgI3 NPs(RLI3). In AgI2 NPs(RLI2), C–C–O, C–C, C–H peaks are narrower. For Ag NPs synthesized from unirradiated and irradiated RF extracts the intensity of C–C–O, coupled O–H, C–H are reduced and broadened in all Ag NPs. However, C–C, C–H, and O–H groups appear as intense peaks in AgI2 NPs(RFI2) and AgI3 NPs(RFI3). Among the functional groups, O–H peaks show a comparatively large shift in both unirradiated and irradiated RL and RF extract synthesized Ag NPs. FTIR spectra conclude that for the synthesis of Ag NPs, functional groups such as C–C–O, coupled O–H and C–H, C–C and O–H groups are largely responsible for the reduction and/or capping of Ag NPs.

It can be seen from FTIR spectra that all the observed functional groups participate in the synthesis of Ag NPs. As observed, most of the functional groups present variation in peak intensity and shift in the position upon irradiation of the extracts and subsequent synthesis of Ag NPs causing change in chemical environment and the intermolecular strength of the functional groups. The modification in the functional group upon irradiation leads to differently shaped and sized NPs as observed in the present study. The anisotropy in shape, their aggregation, and assemblies provide reactive sites for the adsorption of respective analytes and present hotspots for enhancing the Raman signal. The SERS activity of these Ag NPs is discussed in the following section.

## Application in SERS sensing

**Fig. 8:**
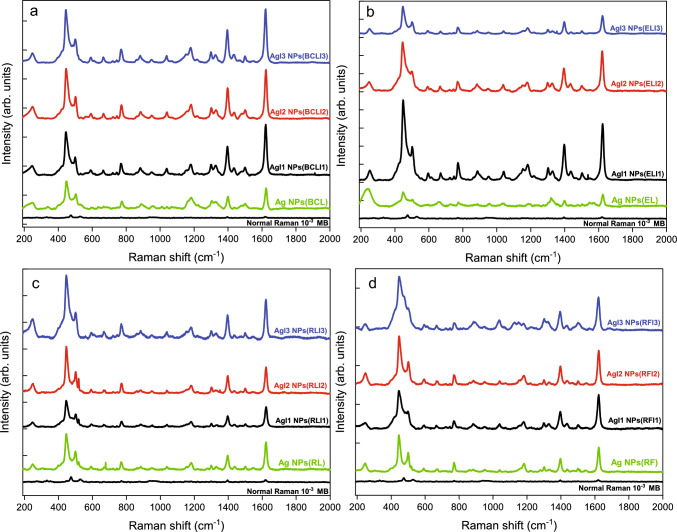
Normal Raman spectrum of 10$^{-3}$ M MB on silicon substrate and SERS Spectra of 10$^{-6}$ M MB on Ag NPs, AgI1 NPs, AgI2 NPs, and AgI3 NPs substrates synthesized using unirradiated and irradiated **a** BCL, **b** EL, **c** RL, and **d** RF extracts

The synthesized Ag NPs using unirradiated and irradiated extracts were applied as SERS substrates to probe the presence of methylene blue (MB) dye in water. SERS was carried on Ag NPs substrates for different concentrations 10$^{-5}$ to 10$^{-9}$ M of MB. Figure 8a,b,c and d shows normal Raman spectra of 10$^{-3}$ M of MB on a silicon substrate and SERS spectra of 10$^{-6}$ M of MB on Ag NPs substrates synthesized by using unirradiated and irradiated BCL, EL, RL, and RF extracts respectively. Raman spectrum of MB show characteristic peaks at 446 cm$^{-1}$, 498 cm$^{-1}$, 1394 cm$^{-1}$ and 1624 cm$^{-1}$ corresponding to C-N-C skeletal deformation vibration, C-N symmetrical stretching and C–C ring stretching bands respectively. The SERS spectrum of MB on Ag NPs substrates presents enhancement of multiple characteristic peaks of MB. A peak at 249 cm$^{-1}$ attributed to Ag-O stretching mode originating from the substrate is also observed [19]. SERS enhancement factor (EF) is evaluated only for the two most intense peaks of MB at 446 cm$^{-1}$ and 1624 cm$^{-1}$ and are given in the supplementary Table S7. An EF of 10$^4$-10$^5$ is obtained while probing 10$^{-6}$ M of MB on these Ag NPs substrates irrespective of irradiation. Referring to Tables S7 and S8, SERS in Ag NPs synthesized from unirradiated BCL, EL, RL, and RF extracts provide a maximum EF of 10$^{6}$. However, upon irradiation, the EF on AgI1 NPs(BCLI1) and AgI1 NPs(ELI1) substrates increases to 10$^7$. Having observed the high EF, the contribution of the plant metabolites in the SERS spectrum is ignored because of two reasons; 1. the SERS substrates were cleaned using repeated centrifugation process prior to their use as sensors and 2. the obtained EF is of the order of 10$^6$-10$^7$ which is purely an electromagnetic enhancement where the contributions from phytomolecules are nearly insignificant. The enhancement to SERS signal appears via two mechanisms; electromagnetic enhancement and chemical enhancement. Electromagnetic enhancement results from amplification of the light by the excitation of LSPR in plasmonic nanomaterials like silver and gold. When molecules are in close proximity to these nanomaterials, their Raman scattering is greatly enhanced due to the increased electromagnetic field intensity. This amplification is mostly concentrated in nanoscale aggregates with narrow inter-particle gaps, and sharp features which offer SERS hotspots. Owing to electromagnetic enhancement SERS signals can be enhanced by factors of $\sim$10$^{10}$-10$^{11}$. The chemical enhancement involves charge transfer mechanisms, between the substrate and probe molecules. Here, the excitation wavelength resonantes with the metal-molecule charge transfer electronic states and offer SERS enhancement factors up to 10$^3$ [1, 11].

**Fig. 9:**
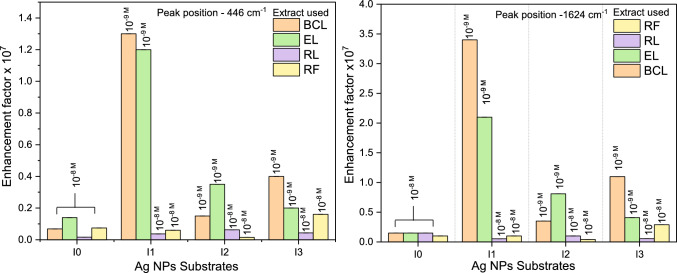
The maximum EFs obtained for MB peaks at 446 cm$^{-1}$ and 1624 cm$^{-1}$ on Ag NPs(I0), AgI1 NPs(I1), AgI2 NPs(I2), and AgI3 NPs(I3) substrates synthesized using unirradiated and irradiated BCL, EL, RL, and RF extracts while probing MB at the lowest concentration (limit of detection 10$^{-8}$/10$^{-9}$ M MB). Here, I0 is the sample code for unirradiated extract

The major contribution to SERS EF is ascribed to the electromagnetic enhancement mechanism, which results from the amplification of the light by the excitation of LSPRs in metallic NPs. Owing to the broad and intense LSPR of the synthesized Ag NPs as observed from UV–vis absorption spectra, the SERS enhancement signal could be achieved. The limit of detection (LOD) of MB was achieved up to 10$^{-9}$M on Ag NPs substrates synthesized using irradiated BCL and EL extracts for all fluences. The LOD is limited to 10$^{-8}$ M for the rest of the Ag NPs synthesized using unirradiated extracts and irradiated RL and RF extracts. The SERS spectra of MB (10$^{-5}$ M to 10$^{-9}$ M) on Ag NPs substrates synthesized using unirradiated and irradiated extracts have been given in the supplementary file. The maximum EFs and the best LOD obtained for the Ag NPs substrates are given in Fig. 9. Amongst these substrates, highest EF of 10$^{7}$ is obtained only for AgI1 NPs(BCLI1), AgI1 NPs(ELI1) for both 446 cm$^{-1}$ and 1624 cm$^{-1}$ peaks of MB. The enhancement depends on the electromagnetic field provided by the substrate. As the electromagnetic field is not uniformly distributed but spatially localized in hotspots that appear at intraparticle voids. The anisotropic shapes of NPs and their large aggregation/clusters in AgI1 NPs(BCLI1), and AgI1 NPs(ELI1) as seen from TEM are responsible for the high observed SERS EFs [65, 66]. The EFs in both 446 cm$^{-1}$ and 1624 cm$^{-1}$ MB peaks as well as the LOD of MB on all Ag NPs substrates synthesized using unirradiated and irradiated extracts have been tabulated in Table S7 and S8.

Due to variations in MB adsorption and their interaction with the electromagnetic field at different sites in the substrate, the obtained EFs reveal non-uniform SERS activity at different sites. To check the reproducibility and homogeneity of SERS activity on Ag NPs, 10$^{-6}$ M of MB adsorbed substrates were scanned at different sites, and their relative standard deviation (RSD) in EF were also examined (see supplementary file). The SERS signal was reproducible on all Ag NPs substrates. However, on Ag NPs synthesized using unirradiated and irradiated BCL extracts, better uniformity in SERS signal at different sites was obtained only for AgI1 NPs(BCLI1) substrate. As a result, AgI1 NPs(BCLI1) substrates present RSD in EFs, 9% and 4% for peaks at 446 cm$^{-1}$ and 1624 cm$^{-1}$ of MB respectively. The RSD is lower in AgI1 NPs(BCLI1) followed by Ag NPs(BCL), AgI3 NPs(BCLI3), and AgI2 NPs(BCLI2) substrates. The high EF of 10$^7$ and uniform SERS on AgI1 NPs(BCLI1) substrates are attributed to their aggregation and homogeneous adsorption of MB. Similarly, for Ag NPs synthesized from unirradiated and irradiated EL, RL, and RF extracts, lowest RSD in EF are obtained for AgI3 NPs(ELI3)(5% and 12%), AgI3 NPs(RLI3)(6% and 8%), and AgI3 NPs(RFI3)(7%) for MB peaks at 446 cm$^{-1}$ and 1624 cm$^{-1}$ respectively. Despite showing relatively lower EF(10$^5$-10$^6$) AgI3 NPs(ELI3), AgI3 NPs(RLI3), and AgI3 NPs(RFI3) substrates are best suited for SERS activity due to uniform adsorption of analyte molecule and homogeneity in SERS spectra over these substrates as compared to other Ag NPs synthesized using unirradiated and irradiated EL, RL and RF extracts. Owing to uniformity in shape, size, and well-dispersed NPs as confirmed by TEM.

The impact of irradiation on SERS transpires in terms of EF and uniformity of SERS signal over the Ag NPs. The anisotropic shapes, aggregation, and intense LSPR provide an electromagnetic enhancement of MB dye signal with EF of order 10$^6$ for all Ag NPs substrates synthesized from unirradiated plant extracts with LOD of 10$^{-8}$ M. SERS spectra on Ag NPs substrates synthesized from BCL and EL extracts show that irradiation leads to 10-fold increase in EF to 10$^7$ on AgI1 NPs(BCLI1) and AgI1 NPs(ELI1), owing to the presence of anisotropic shapes and large aggregation in these Ag NPs which offer localized hotspots for electromagnetic enhancement. Further, an improved LOD of 10$^{-9}$ M is obtained for MB on AgI1 NPs, AgI2 NPs, and AgI3 NPs substrates synthesised using irradiated BCL and EL extracts. The EF of SERS spectra on Ag NPs substrates synthesized from unirradiated and irradiated RL extracts is limited to 10$^6$ with LOD 10$^{-8}$ M for all Ag NPs due to presence of oxide layer of similar thickness and very few aggregation. Ag NPs substrates synthesized from unirradiated and irradiated RF extracts are uniform with sparse aggregation and absence of oxide layer but it also presents EF of 10$^6$ with LOD 10$^{-8}$ M. This appears due to absence of localized hotspots for electromagnetic enhancement which often occurs at interparticle spacing or edges. The irradiation experiment was performed on thin film of the extract of thickness $\sim$74 $\mu$m which not only allowed for the uniform modification of phytomolecules but also led to uniform changes in the synthesised Ag NPs. Despite the morphologies observed in TEM are the localised properties of the sample, we presume them to be extendable to the ensemble. Our presumption is evident from the SERS analysis where the change in morphology such as anisotropic shapes and aggregation of the Ag NPs give rise to uniform electromagnetic enhancement throughout the sample. The observed EF of 10$^6$-10$^7$ while probing MB is better or at par with EF obtained on similar substrates fabricated with high-cost nanolithography-etching-deposition process, laser ablation, composited with other materials such as reduced graphene oxide, multiwalled carbon nanotubes and 3D graphene foam [21, 30, 31, 67, 68]. The EF reported in this work also surpasses that of differently shaped Ag NPs substrates like cap-shaped, nanosphere/nanoplates, triangular, hexagonal, and quasi-spherical nanoparticles, and green synthesized self-assembled nanostructures [19, 23, 69].

## Conclusion

Ag NPs have been synthesized via green synthetic route using unirradiated and irradiated BCL, EL RL, and RF extracts while keeping the synthesis parameters constant. 200 MeV, Ag$^{15+}$ ion irradiation is used to alter the phytomolecules in the plant extract. The impact of irradiation is seen on C–O–C, C–C–O, coupled O–H and C–H, C–C, C–H, and O–H functional groups as a change in intermolecular strength and chemical environments of the organic molecules. Further, these alterations directly impact their participation in the synthesis, as reducing/capping agents and also towards surface functionalization of Ag NPs. Owing to this, different morphologies and sizes of Ag NPs have been observed. Owing to the changes in surface functionalization of these NPs, they present defects due to surface oxidation and aggregation. With respect to obtained morphologies, sizes, and surface oxidation in the synthesized Ag NPs it is drawn that among the utilized plant extracts unirradiated and irradiated RF extracts presents better control as reducing agent giving rise to uniformity in size distribution and morphology.

The NPs synthesized using unirradiated and irradiated extracts are utilized as SERS substrates to detect MB. An electromagnetic enhancement of MB dye signal with EF of order 10$^6$-10$^7$ with LOD of MB up to nanomolar concentration is obtained using these Ag NPs substrates. The anisotropic shapes, aggregation, and intense LSPR are attributed to the observed SERS activity which are better than or at par with those Ag NPs synthesized via other green/chemical techniques. Of all the examined SERS substrates, in this work, AgI1 NPs(BCLI1), AgI3 NPs(ELI3), AgI3 NPs(RLI3), and AgI3 NPs(RFI3) are found to be best suited as SERS substrates due to their high and uniform EF of the analyte molecule with an RSD < 12%. This paper presents the modulation in shapes and sizes of Ag NPs by irradiation of phytoextracts. By considering the initial size of these Ag NPs, the direct effect of irradiation on the morphologies and size of Ag NPs can be proposed.

### Additional file

Supplementary file 1 (pdf 4568 KB)

## Data Availability

All data generated and/or analyzed during this study are available from the corresponding author upon reasonable request.

## Declarations

### Research involving plants statement

Experimental studies and field research on plants (cultivated or wild), including the collection of plant material, must comply with relevant institutional, national and international guidelines and legislation. We strictly adhere to the IUCN Policy Statement on Research on Endangered Species and the Convention on Trade in Endangered Species of Wild Fauna and Flora. We confirm compliance with the IUCN policy for plant.
