# Supplementary material for: Refining shape and size of silver nanoparticles using ion irradiation for enhanced and homogeneous SERS activity
Source: Discov Nano. 2024 Mar 19;19(1):51. doi: 10.1186/s11671-024-03994-x (PMC11329486; doi:10.1186/s11671-024-03994-x)
Supplement: Supplementary file 1 — (pdf 4568 KB) [file 11671_2024_3994_MOESM1_ESM.pdf]

Refining shape and size of silver nanoparticles using ion irradiation for enhanced and homogeneous SERS activity

Laden Sherpa[1], Arun Nimmala[2], S.V.S Nageswara Rao[2], S.A Khan[3], Anand P Pathak[4], Ajay Tripathi[1], Archana Tiwari[5]

[1]Department of Physics, Sikkim University, Tadong, Gangtok, 737102, Sikkim, India.
[2]Centre for Advanced Studies in Electronics Science and Technology (CASEST), School of Physics, University of Hyderabad, Hyderabad,500046, Telangana,India.
[3]Inter University Accelerator Centre, (IUAC), New Delhi,110067, New Delhi, India.
[4]School of Physics University of Hyderabad, Hyderabad, 5000046, Telangana, India.
[5]Department of Physics, Institute of Science, Banaras Hindu University, Varanasi, 221005, Uttar Pradesh, India.

Corresponding authors: archana.tiwari.ox@gmail.com;

Keywords: Ion irradiation, Green synthesis, SERS, Enhancement factor

Supporting Information

0.1 Deconvolution of UV-vis absorption and PL peaks

UV-vis absorption spectra of Ag NPs synthesized using unirradiated and irradiated BCL and EL extracts are shown in Fig.S1. Here, the broad absorption peaks are deconvoluted and the obtained peak positions have been labeled. UV-vis absorption spectra of Ag NPs synthesized using unirradiated and irradiated RL and RF extracts and their deconvolution are shown in Fig.S2 where the obtained peak positions have been labeled.

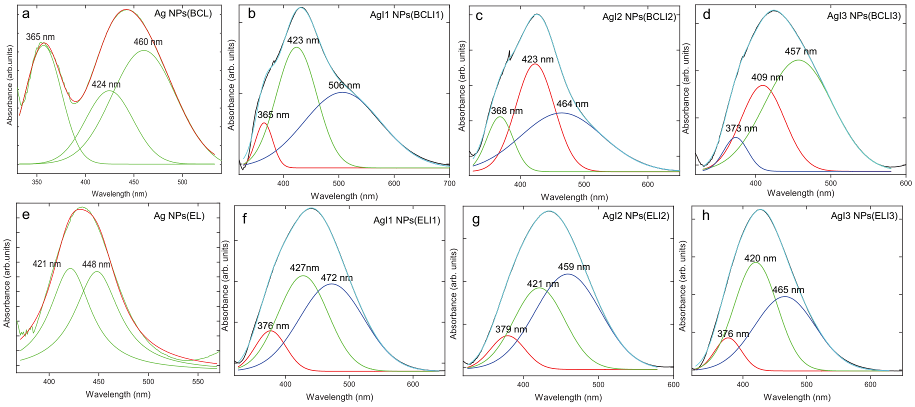

Fig. S1 UV-Vis absorption spectra and deconvolution of peaks in a. Ag NPs(BCL), b. AgI1 NPs(BCLI1), c. AgI2 NPs(BCLI2), d. AgI3 NPs(BCLI3), e. Ag NPs(EL), f. AgI1 NPs(ELI1), g. AgI2 NPs(ELI2), h. AgI3 NPs(ELI3).

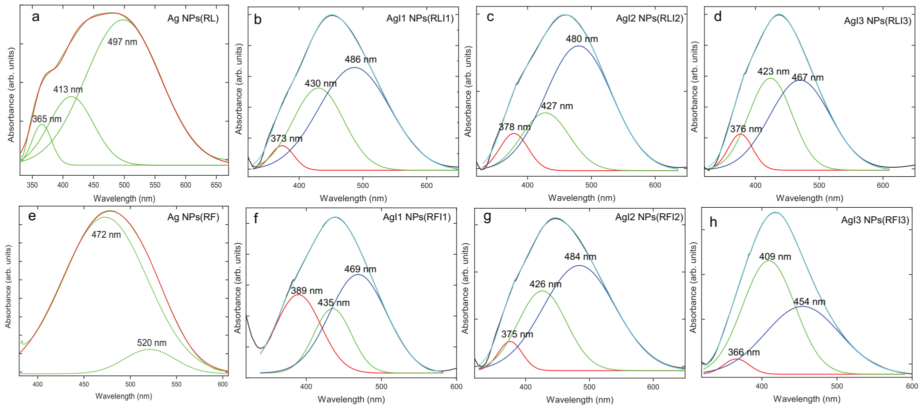

Fig. S2 UV-Vis absorption spectra and deconvolution of peaks in a. Ag NPs(RL), b. AgI1 NPs(RLI1), c. AgI2 NPs(RLI2), d. AgI3 NPs(RLI3), e. Ag NPs(RF), b. AgI1 NPs(RFI1), c. AgI2 NPs(RFI2), d. AgI3 NPs(RFI3).

PL and PLE spectra of Ag NPs synthesized using unirradiated BCL, EL, RL, and RF extracts are given in Fig.S3. The deconvolution and the respective peaks have been labeled in the figure.
PL spectra and deconvolution of PL peaks in Ag NPs synthesized using irradiated BCL, EL, RL, and RF extracts are shown in Fig.S4 and Fig.S5. The deconvoluted peaks have been labeled in the figure.

0.2 Size and morphology of synthesized Ag NPs using unirradiated and irradiated extracts

The obtained morphology, size, and standard deviation in size distribution histogram of Ag NPs synthesized using unirradiated and irradiated BCL, EL, RL, and RF extracts are given in Table S1. It can be seen from the table that upon irradiation of

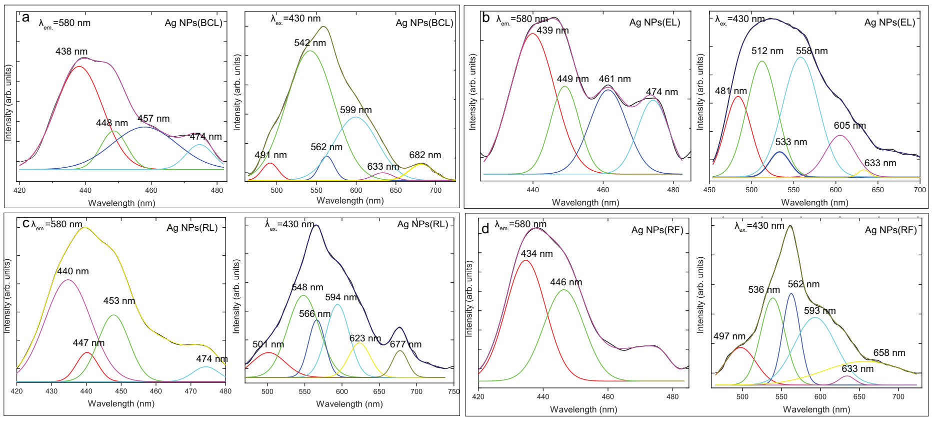

Fig. S3 PL, PLE spectra and deconvolution of peaks in a. Ag NPs(BCL), b. Ag NPs (EL), c. Ag NPs(RL), d. Ag NPs(RF).

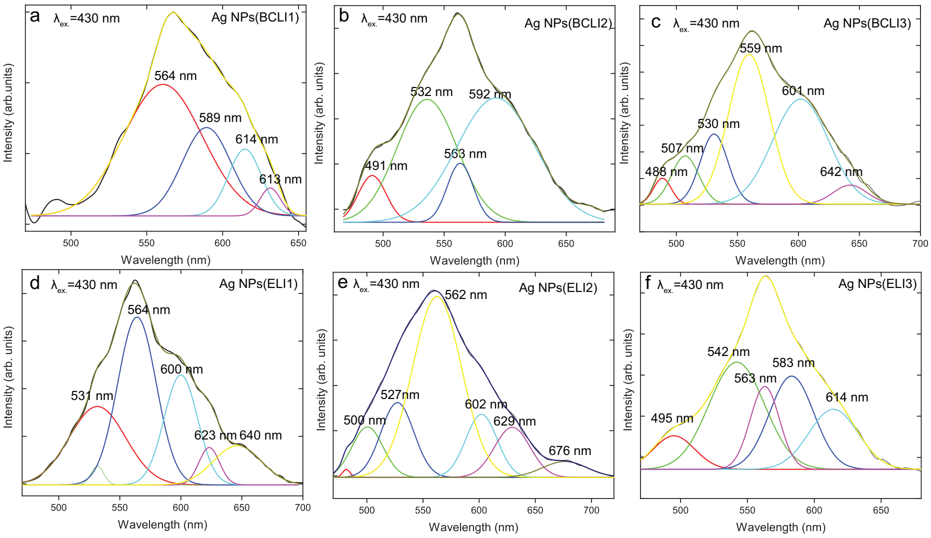

Fig. S4 PL, PLE spectra and deconvolution of peaks in a. AgI1 NPs(BCLI1), b. AgI2 NPs(BCLI2), c. AgI3 NPs(BCLI3), d. AgI1 NPs(ELI1), e. AgI2 NPs(ELI2) f. AgI3 NPs(ELI3)

the extracts the subsequent synthesis of Ag NPs leads to change in the particle size and shapes. Interestingly, in the case of Ag NPs synthesized using BCL extracts the particle size increases with increase in irradiation fluence. However, the size of Ag NPs synthesized using unirradiated and irradiated BCL, EL, RL, and RF extracts are comparable. Further, the standard deviation(σ) in Gaussian fit of size distribution reveals that the uniformity in size is observed for Ag NPs synthesized using BCLI2, ELI3, RLI1 extracts. Moreover, the size distribution is mostly homogeneous for Ag NPs synthesized using unirradiated and irradiated RF extracts with $\sigma \sim 5$ nm.

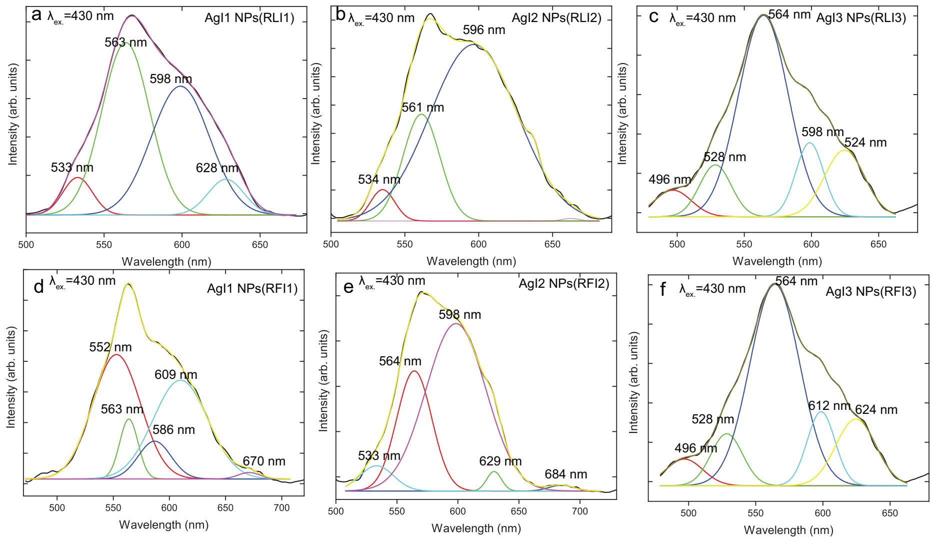

Fig. S5 PL, PLE spectra and deconvolution of peaks in a. AgI1 NPs(RLI1), b. AgI2 NPs(RLI2), c. AgI3 NPs(RLI3), d. AgI1 NPs(RFI1), e. AgI2 NPs(RFI2) f. AgI3 NPs(RFI3)

1 Crystallite size calculation

The crystallite size(D) of synthesized Ag NPs is calculated using Debye-Scherrer equation.

$$D = \frac{\kappa\lambda}{\beta cos\theta} \tag{1}$$

where the Scherrer constant κ=0.94, wavelength of X-ray Cu Kα radiation λ=1.54Å, θ is the Braggs angle and β is the full-width half maximum of intense XRD peaks of both Ag and Ag_2O phases. Ag NPs synthesized from unirradiated extracts exhibit Ag Phases, except for Ag NPs(RF) where both Ag and Ag_2O phases are seen. However, Ag NPs synthesized from irradiated BCL, EL, RL, and RF extracts show both Ag and Ag_2O phases as the irradiation of extract introduces oxidation in these Ag NPs. The calculated crystallite size for Ag and Ag_2O phases are given in TableS2.

1.1 Tauc Plot and schematic of energy manifolds in Ag NPs

The optical band edge is estimated with the help of Tauc plot using the following equation

$$(\alpha h\nu)^N = A(h\nu - E_g) \tag{2}$$

where α is the absorption coefficient, h is Planck's constant, ν is the frequency of the photon, E_g is the bandgap and A is a constant of proportionality. The exponent term N denotes the nature of the electronic transition where N= 2 and 1/2 corresponds to direct and indirect allowed transitions. N=2 for direct transition have been utilized in order to evaluate direct band-gap in these Ag NPs. The estimated band-gap for all Ag NPs are given in Fig.S6.

A schematic of the energy manifold for Ag NPs synthesized using unirradiated and

Table S1 Details of morphology, average particle size fitted with Gaussian distribution, standard deviation(σ) in size distribution, and shell thickness of oxide layer in Ag NPs synthesized using unirradiated and irradiated plant extracts.

Ag NPs	Morphology	Particle size	σ	Shell thickness
Ag NPs(BCL)	quasispherical, elongated, nanorods*	18 nm, 10(D)*, 71(L)*	7 nm	-
AgI1 NPs(BCLI1)	spherical, quasispherical, trigonal, hexagonal	18 nm	7 nm	3.2 nm
AgI2 NPs(BCLI2)	spherical, quasispherical	22 nm	5 nm	3 nm
AgI3 NPs(BCLI3)	spherical, dendrites	23 nm	8 nm	-
Ag NPs(EL)	spherical, quasispherical, trigonal, hexagonal,cylindrical	19 nm	7 nm	
AgI1 NPs(ELI1)	spherical, quasispherical, elongated, trigonal	15 nm	6 nm	4 nm
AgI2 NPs(ELI2)	spherical, quasispherical, cylindrical,trigonal	19 nm	9 nm	-
AgI3 NPs(ELI3)	spherical, quasispherical	13 nm	5 nm	-
Ag NPs(RL)	spherical, quasispherical, cylindrical	17 nm	8 nm	
AgI1 NPs(RLI1)	spherical, quasispherical, cylindrical	23 nm	5 nm	11 nm
AgI2 NPs(RLI2)	spherical, quasispherical, trigonal, cylindrical	18 nm	10 nm	10 nm
AgI3 NPs(RLI3)	spherical, quasispherical, cylindrical	19 nm	10 nm	10 nm
Ag NPs(RF)	spherical, quasispherical, trigonal, cylindrical	17 nm	5 nm	
AgI1 NPs(RFI1)	spherical, quasispherical, elongated	24 nm	5 nm	-
AgI2 NPs(RFI2)	spherical, quasispherical, hexagonal, trigonal	20 nm	6 nm	-
AgI3 NPs(RFI3)	cylindrical, spherical, porous	18 nm	5 nm	-

irradiated extracts is been shown in Fig. S7 by using estimated band-gap and PL energies. The visible luminescence includes transitions from states below the band-edge revealing the presence of defect states in the Ag NPs.

1.2 Mechanism of Ag NPs synthesis

The reduction mechanism of Ag ions utilizing some of the polyphenolic compounds such as epicatechin (a phenol) and quercetin (a flavonoid) present in the unirradiated BCL extract is shown in Fig.S8. The reduction may also take place simultaneously by utilizing different polyphenols that provide stability against agglomeration. In a

Table S2 Intense peak position recorded for Ag and Ag_2O crystalline phases using XRD. Their corresponding (hkl) planes and crystallite size calculated using Debye-Scherrer equation.

Ag/Ag_2O NPs	Phase	$2\theta(°)$	Crystal plane	Crystallite size(nm)
Ag NPs(BCL)	Ag	38.1	(111)	6.2 nm
AgI1 NPs(BCLI1)	Ag	38.1	(111)	6.9 nm
AgI2 NPs(BCLI2	Ag_2O	32.0	(111)	3.9nm
	Ag	38.0	(111)	4.8 nm
AgI3 NPs(BCLI3)	Ag_2O	32.2	(111)	4.5 nm
	Ag	38.2	(111)	4.1 nm
Ag NPs(EL)	Ag	37.9	(111)	5.5 nm
AgI1 NPs(ELI1)	Ag_2O	32.3	(111)	6.4 nm
	Ag	38.1	(111)	6.5 nm
AgI2 NPs(ELI2	Ag_2O	32.2	(111)	6.9 nm
	Ag	38.1	(111)	4.8 nm
AgI3 NPs(ELI3)	Ag_2O	32.0	(111)	6.4 nm
	Ag	37.7	(111)	3.8 nm
Ag NPs(RL)	Ag	38.1	(111)	4.9 nm
AgI1 NPs(RLI1)	Ag_2O	32.2	(111)	7.5 nm
	Ag	38.1	(111)	4.9 nm
AgI2NPs(RLI2	Ag_2O	32.1	(111)	10 nm
	Ag	37.9	(111)	6.8 nm
AgI3 NPs(RLI3)	Ag_2O	32.2	(111)	6.9 nm
	Ag	38.2	(111)	6.4 nm
Ag NPs(RF)	Ag_2O	32.4	(111)	11.2 nm
	Ag	38.2	(111)	4.8 nm
AgI1 NPs(RFI1)	Ag_2O	32.3	(111)	7.9 nm
	Ag	38.1	(111)	5.3 nm
AgI2 NPs(RFI2)	Ag_2O	32.1	(111)	9.4 nm
	Ag	37.9	(111)	7.7 nm
AgI3 NPs(RFI3)	Ag_2O	32.2	(111)	11.0 nm
	Ag	38.1	(111)	6.8 nm

similar manner, phytomolecules present in EL, RL, and RF extract may participate in the reduction and/or capping of Ag NPs.

1.3 FTIR spectra and the assignments of functional groups

FTIR peak positions and the assigned functional groups in Ag NPs synthesized using unirradiated and irradiated BCL, EL, RL and RF extract is given in Table S3,S4,S5,S6.

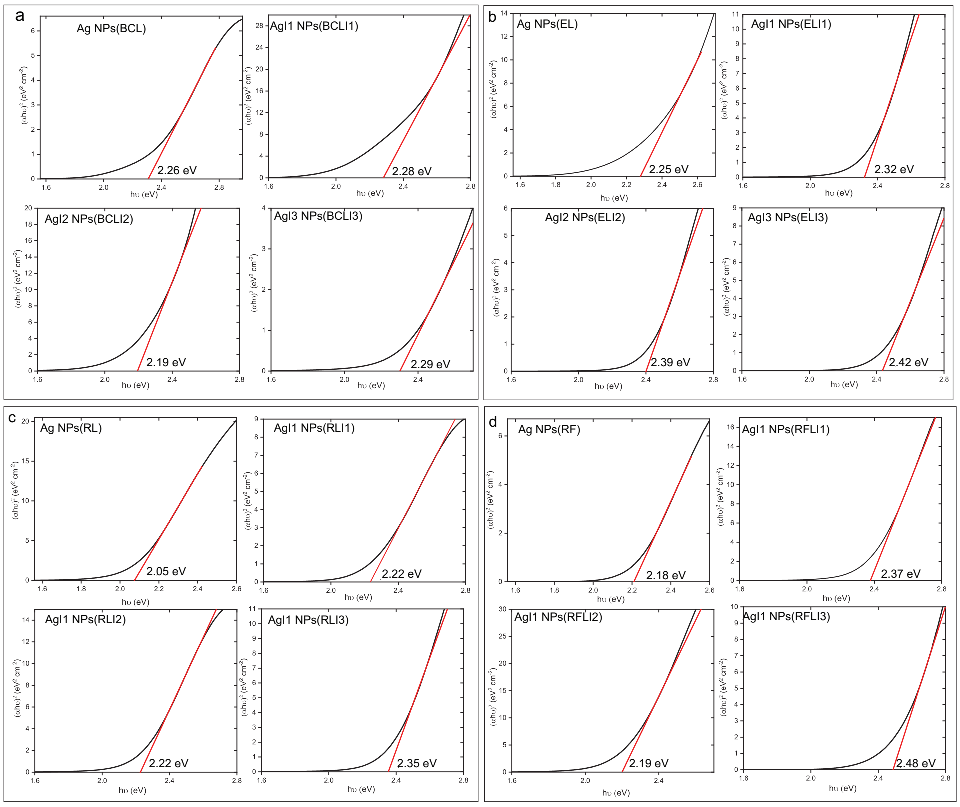

Fig. S6 Tauc plots of Ag NPs synthesized using unirradiated and irradiated a. BCL b. EL, c. RL and d. RF extracts.

1.4 SERS activity of Ag NPs substrates

The identification, detection, and qualitative determination of methylene blue (MB) molecules in water was performed using SERS. Ag NPs synthesized using unirradiated and irradiated BCL, EL, RL, and RF extracts were used as substrates to probe different concentrations (10^{-5} M to 10^{-9} M) of MB. Raman signal of MB on these substrates and a normal Raman spectrum of 10^{-3} M MB on silicon substrate were recorded. Two intense peaks of MB at 446 cm^{-1} and 1624 cm^{-1} were selected for calculation of enhancement factor (EF). The EF in Raman peaks is defined as

$$EF = \frac{I_{SERS}}{I_{Raman}} \times \frac{N_{Raman}}{N_{SERS}} \tag{3}$$

where I_{SERS}, I_{Raman} are Raman peak intensities and N_{SERS}, N_{Raman} are concentrations of MB used for SERS activity on Ag NPs substrate and normal Raman (10^{-3} M MB) on silicon substrate respectively. The SERS spectra MB (10^{-5} M to 10^{-9}

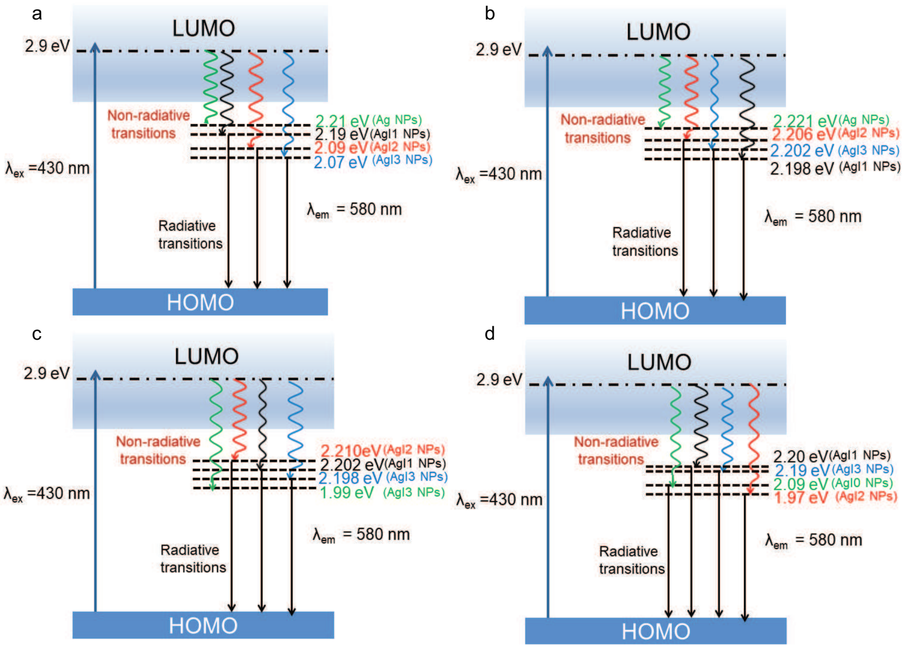

Fig. S7 A schematic of energy level diagram in Ag NPs synthesized using unirradiated and irradiated a. BCL b.EL, c. RL and d. RF extracts.

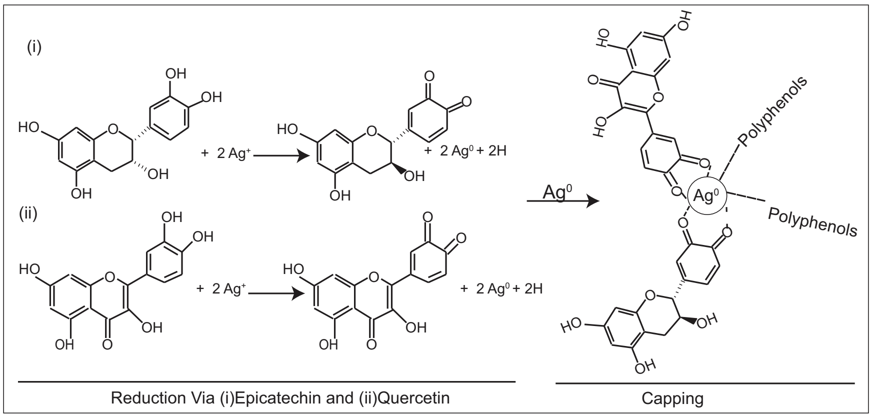

Fig. S8 A schematic of reduction mechanism of synthesis of Ag NPs(BCL) using phytomolecules present in BCL extract.

M)on all Ag NPs substrates, reproducibility at 10^{-6} M, and relative standard deviation in the enhancement factor is shown in Fig.S9. SERS spectra of MB on Ag NPs substrates synthesized using unirradiated and irradiated BCL extracts are shown in Fig. S9. Here I0 in the figures represents the unirradiated condition of the extracts. SERS signal of MB was obtained for 10^{-5} M to 10^{-8} M MB on AgI0 NPs(BCLI0)

Table S3 FTIR peaks obtained from unirradiated and irradiated BCL extracts, as-synthesized Ag NPs, and their assignment with the corresponding functional groups.

Assigned functional groups	FTIR peak positions in cm^{-1}							
	Extracts				Nanoparticles			
	BCL	BCLI1	BCLI2	BCLI3	Ag	AgI1	AgI2	AgI3
C-O-C asymmetric stretch	959	958	961		962			
C-C-O stretch in phenols	1082<br>1141<br>1252	1078<br>1155<br>1228	1050<br>1133<br>1254	1092<br>1140<br>1260	1083<br>1147<br>1264	1089<br>1135<br>1225	1089<br>1138<br>1206	1091<br>1135<br>1243
O-H in-plane bending, C-H wagging in primary, secondary alcohols	1399<br>1442	1373<br>1420	1368<br>1415	1364<br>1487	1387<br>1455	1353<br>1423	1394<br>1427	1370<br>1404
C=O carbonyl stretch	1513		1512	1507			1513	1505
C-C aromatic stretch	1597	1613	1598	1604	1578	1620	1587	1600
C-H stretch in aromatics, alkanes	2859<br>2935	2942	2771<br>3021	2933	2853<br>2930	2955	2934	2927
O-H stretch in alcohols, phenols	3362	3393	3400	3381	3377	3353	3363	3361

substrate which increases to 10^{-9} M MB on AgI1 NPs(BCLI1), AgI2 NPs(BCLI2) and AgI3 NPs(BCLI3) substrates. Similarly, SERS enhancement of MB peaks were observed for Ag NPs synthesized using unirradiated and irradiated EL, RL, and RF extracts as shown in Fig.S10,S11, andS12. Upon irradiation of extracts, the limit of detection (LOD) of MB on Ag NPs substrates extends to 10^{-9} M for irradiated BCL and EL extract synthesized Ag NPs. However, LOD is limited to 10^{-8} M on irradiated RL and RF extract synthesized Ag NPs.

To check the reproducibility of SERS signal on Ag NPs substrates, 10^{-6} M MB was chosen and Raman spectra were recorded at 4 different sites. The EF of the MB signal at each site for 446 cm^{-1} and 1624 cm^{-1} peaks and their relative standard deviation (RSD) in the EFs were evaluated. The SERS substrate that offers uniform SERS signal enhancement is obtained for AgI1 NPs(BCLI1), AgI3 NPs(ELI3), AgI3 NPs(RLI3), and AgI3 NPs(RFI3) with comparatively low RSD due to homogenous dispersion and size distribution of Ag NPs. In order to highlight the EFs in both 446 cm^{-1} and 1624 cm^{-1} MB peaks as well as the LOD of MB on these Ag NPs substrates have been tabulated in TableS7 andS8.

Table S4 FTIR peaks obtained from unirradiated and irradiated EL extracts, as-synthesized Ag NPs, and their assignment with the corresponding functional groups.

Assigned functional groups	FTIR peak positions in cm^{-1}							
	Extracts				Nanoparticles			
	EL	ELI1	ELI2	ELI3	Ag	AgI1	AgI2	AgI3
C-O-C asymmetric stretch	969	956	956					
C-C-O stretch in phenols	1098<br>1144<br>1240	1092<br>1144	1093<br>1150	1085<br>1137	1085<br><br>1241	1093<br>1138	1093<br>1145	1080<br>1158
O-H in-plane bending, C-H wagging in primary, secondary alcohols	1396<br>1448	1357<br>1455	1364<br>1448	1401<br>1487	1395<br>1448	1357<br>1435	1363<br>1448	1415<br>1486
C-C aromatic stretch	1597<br>1668	1649	1623	1649	1552<br>1649	1634	1647	1648
C-H stretch in aromatics, alkanes	2873<br>2919	2942	2771	2933	2867<br>2932	2955	2934	2927
O-H stretch in alcohols, phenols	3385	3236	3379	3373	3301	3240	3343	3356

Table S5 FTIR peaks obtained in unirradiated and irradiated RL extracts, as-synthesized Ag NPs and their assignment with the corresponding functional groups.

Assigned functional groups	FTIR peak positions in cm^{-1}							
	Extracts				Nanoparticles			
	RL	RLI1	RLI2	RLI3	Ag	AgI1	AgI2	AgI3
C-O-C asymmetric stretch	962				964	956		
C-C-O stretch in phenols	1086<br>1139<br>1290	1092<br>1144	1079<br>1150	1085<br>1150	1092<br>1137<br>1286	1079<br>1145<br>1286	1079<br>1137	1085<br><br>1215
O-H in-plane bending, C-H wagging in primary, secondary alcohols	1396<br>1436	1357<br>1423	1360<br>1448	1364<br>1429	1390<br>1452	1353<br>1442	1357<br>1448	1396<br>1446
C-C aromatic stretch	1617	1617	1629<br>1713	1619	1618	1610	1617<br>1713	1611
C-H stretch in aromatics, alkanes	2845<br>2936	2925	2930	3074	2854<br>2925	2926	2919	2912
O-H stretch in alcohols, phenols	3384	3253	3392	3405	3373	3334	3366	3360

Table S6 FTIR peaks obtained in unirradiated and irradiated RF extracts, as-synthesized Ag NPs, and their assignment with the corresponding functional groups.

Assigned functional groups	FTIR peak positions in cm^{-1}							
	Extracts				Nanoparticles			
	RF	RFI1	RFI2	RFI3	Ag	AgI1	AgI2	AgI3
C-O-C asymmetric stretch	956	953	956		956	957	953	
C-C-O stretch in phenols	1085<br>1144<br>1288	1092<br>1144	1090<br>1144	1079<br>1137	1087<br>1137<br>1286	1092<br>1142	1094<br>1140	1090<br>1144
O-H in-plane bending, C-H wagging in primary, secondary alcohols	1383<br>1448	1364<br>1442	1357<br>1436	1364<br>1430	1386<br>1436	1354<br>1448	1351<br>1446	1361<br>1401
C-C aromatic stretch	1615	1617	1623	1604	1617	1604	1617	1598
C-H stretch in aromatics, alkanes	2855<br>2944	2944	2940	2932	2848<br>2932	2938	2925	2928
O-H stretch in alcohols, phenols	3360	3253	3327	3314	3353	3327	3366	3314

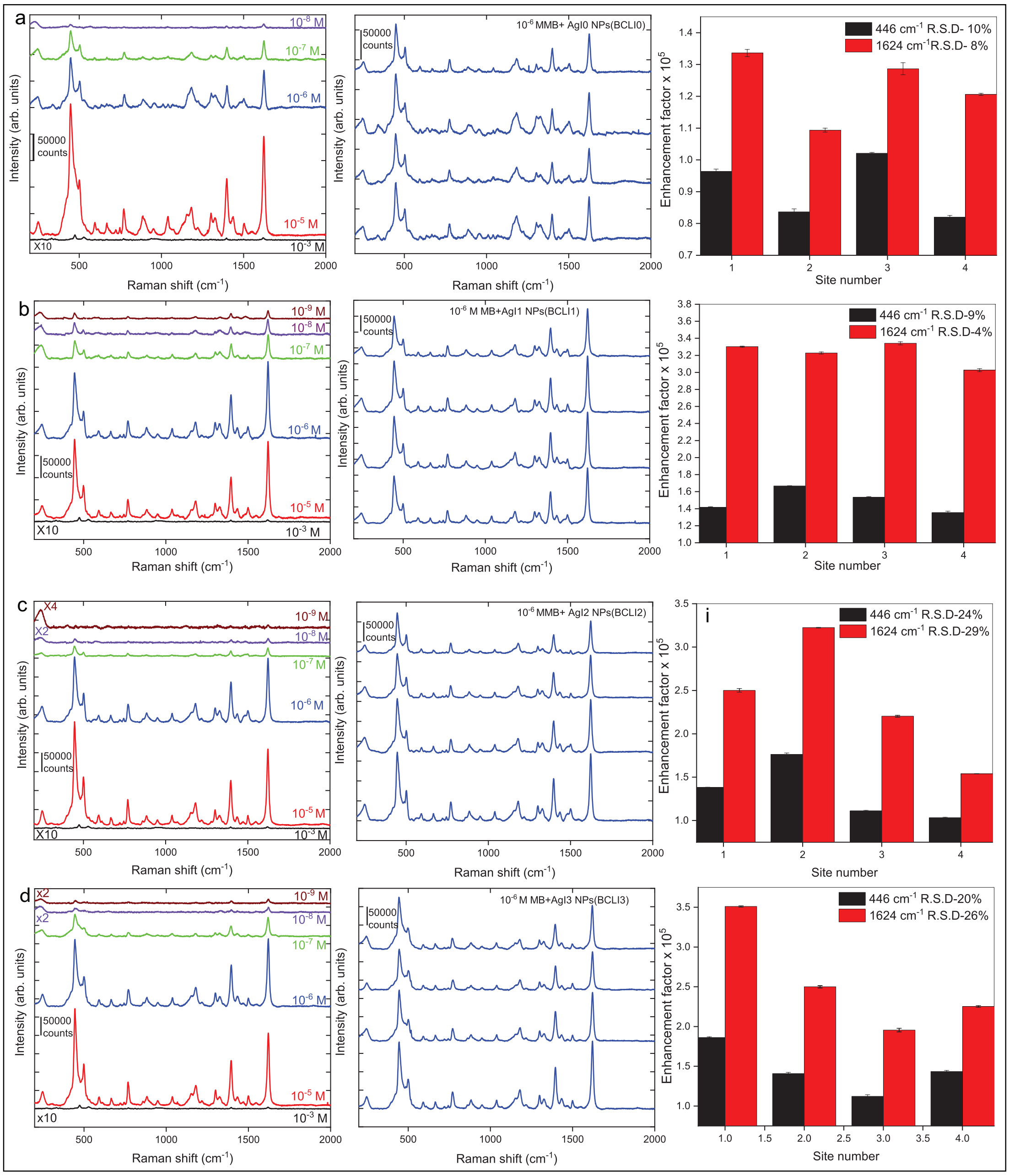

Fig. S9 Raman spectrum of MB (10^{-3} M), SERS spectra of 10^{-5} to $10^{-8}/10^{-9}$ M MB, reproduced SERS spectra of MB 10^{-6} M MB at different substrate sites, RSD in EF on a. AgI0 NPs(BCLI0) b. AgI1 NPs(BCLI1), c. AgI2 NPs(BCLI2) and d. AgI3 NPs(BCLI3) substrates.

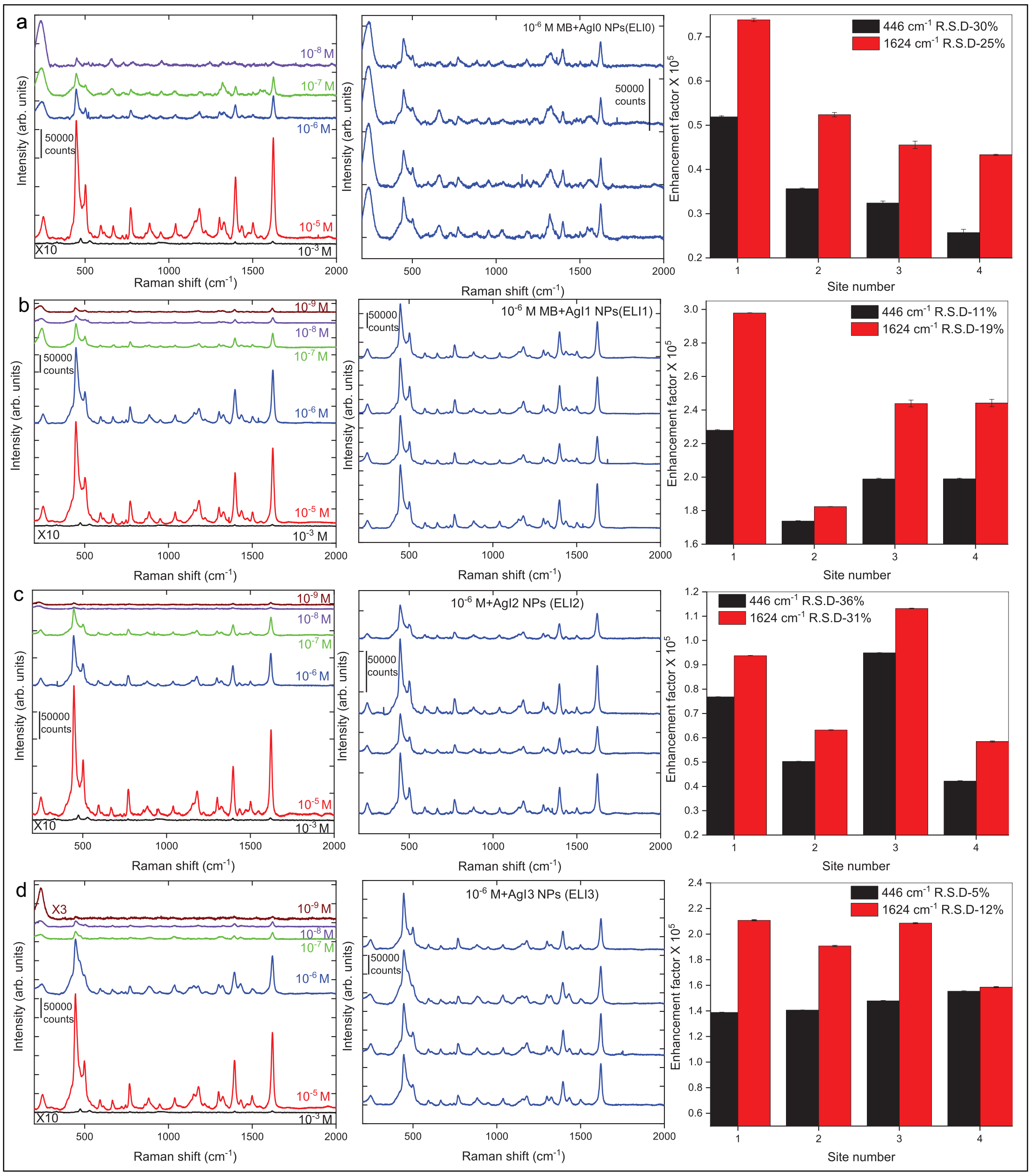

Fig. S10 Raman spectrum of MB (10^{-3} M), SERS spectra of 10^{-5} to $10^{-8}/10^{-9}$ M MB, reproduced SERS spectra of MB 10^{-6} M MB at different substrate sites, RSD in EF on a. AgI0 NPs(ELI0) b. AgI1 NPs(ELI1), c. AgI2 NPs(ELI2) and d. AgI3 NPs(ELI3) substrates.

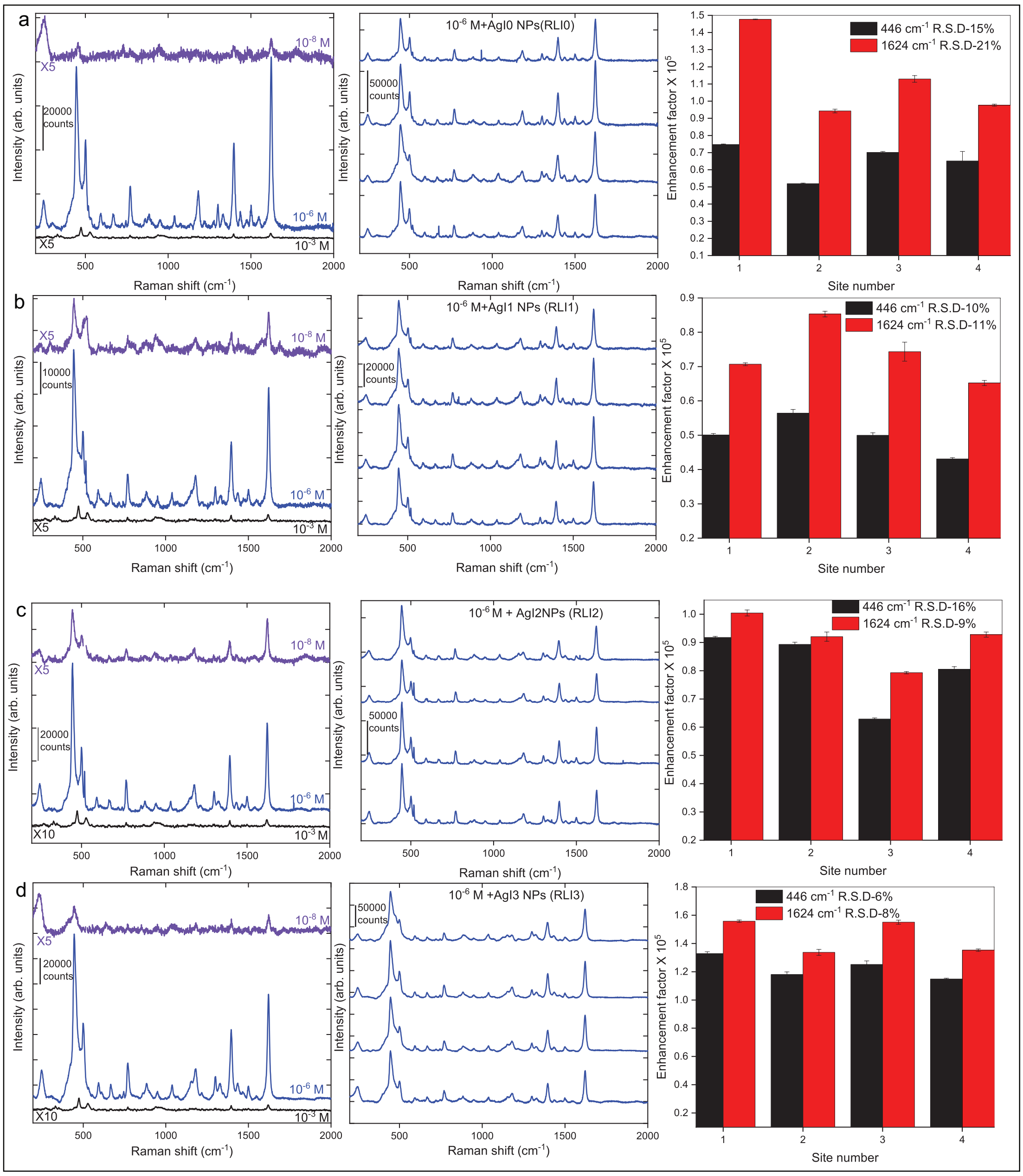

Fig. S11 Raman spectrum of MB (10^{-3} M), SERS spectra of 10^{-5} to 10^{-8} M MB, reproduced SERS spectra of MB 10^{-6} M MB at different substrate sites, RSD in EF on a. AgI0 NPs(RLI0) b. AgI1 NPs(RLI1), c. AgI2 NPs(RLI2) and d. AgI3 NPs(RLI3) substrates.

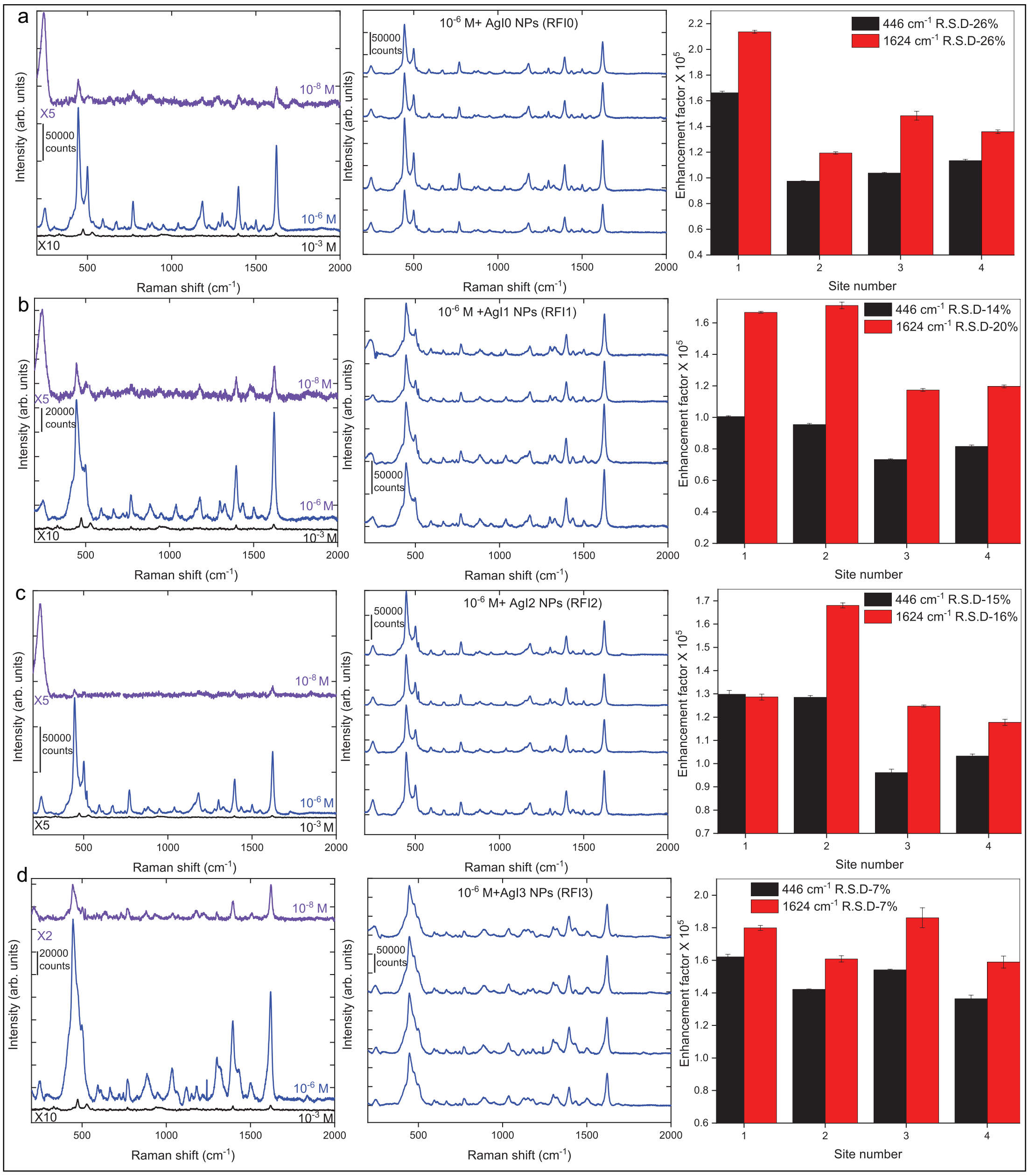

Fig. S12 Raman spectrum of MB (10^{-3} M), SERS spectra of 10^{-5} to 10^{-8} M MB, reproduced SERS spectra of MB 10^{-6} M MB at different substrate sites, RSD in EF on a. AgI0 NPs(RFI0) b. AgI1 NPs(RFI1), c. AgI2 NPs(RFI2) and d. AgI3 NPs(RFI3) substrates.

Table S7 Enhancement factor obtained for MB peaks at 446 cm^{-1} and 1624 cm^{-1}, and the limit of detection of MB on Ag NPs substrates synthesized using unirradiated and irradiated BCL and EL extracts.

Ag NPs substrate	Concentration of MB (Molar)	Enhancement factor in 446 cm^{-1}	Enhancement factor factor in 1624 cm^{-1}	Limit of detection (Molar)
AgI0 NPs(BCLI0)	10^{-5} M	2.6 x10^4	3.6x10^4	10^{-8} M
	10^{-6} M	1.0x10^5	1.3x10^5	
	10^{-7} M	5.6x10^5	6.9x10^5	
	10^{-8} M	6.9x10 ^{5}	1.5x10^6	
	10^{-9} M	-	-	-
AgI1 NPs(BCLI1)	10^{-5} M	1.8x10^4	3.3x10^4	10^{-9} M
	10^{-6} M	1.7x10^5	3.3x10^5	
	10^{-7} M	4.1x10^5	1.0x10^6	
	10^{-8} M	2.6x10^6	6.3x10^6	
	10^{-9} M	1.3x10^7	3.4x10^7	
AgI2 NPs(BCLI2)	10^{-5} M	2.8x10^4	3.8x10^4	10^{-9} M
	10^{-6} M	1.8x10^5	3.2x10^5	
	10^{-7} M	2.7x10^5	4.1x10^5	
	10^{-8} M	5.0x10^5	1.0x10^5	
	10^{-9} M	1.5x10^6	3.5x10^6	
AgI3 NPs(BCLI3)	10^{-5} M	2.7x10^4	3.7x10^4	10^{-9} M
	10^{-6} M	1.8x10^5	3.5x10^5	
	10^{-7}M	6.1x10^5	9.8x10^5	
	10^{-8}M	5.9x10^5	1.3x10^6	
	10^{-9}M	4.0x10^6	1.1x10^7	
AgI0 NPs(ELI0)	10^{-5} M	2.1x10^4	3.4x10^4	10^{-8} M
	10^{-6} M	2.2x10^4	7.4x10^4	
	10^{-7} M	3.9x10^5	5.9x10^5	
	10^{-8} M	1.4x10 ^{6}	1.5x10^6	
	10^{-9} M	-	-	-
AgI1 NPs(ELI1)	10^{-5} M	3.0x10^4	4.1x10^4	10^{-9} M
	10^{-6} M	2.3x10^5	2.9x10^5	
	10^{-7} M	7.1x10^5	7.7x10^5	
	10^{-8} M	2.2x10^6	2.9x10^6	
	10^{-9} M	1.2x10^7	2.1x10^7	
AgI2 NPs(ELI2)	10^{-5} M	2.5x10^4	3.0x10^4	10^{-9} M
	10^{-6} M	9.5x10^4	1.1x10^5	
	10^{-7} M	5.0x10^5	6.3x10^5	
	10^{-8} M	3.4x10^5	5.9x10^5	
	10^{-9} M	3.5x10^6	8.1x10^6	
AgI3 NPs(ELI3)	10^{-5} M	3.1x10^4	3.8x10^4	10^{-9} M
	10^{-6} M	1.4x10^5	2.1x10^5	
	10^{-7}M	1.9x10^5	3.2x10^5	
	10^{-8}M	1.6x10^6	2.5x10^6	
	10^{-9}M	2.0x10^6	4.1x10^6	

Table S8 Enhancement factor obtained for MB peaks at 446 cm^{-1} and 1624 cm^{-1}, and the limit of detection of MB on Ag NPs substrates synthesized using unirradiated and irradiated RL and RF extracts.

Ag NPs substrate	Concentration of MB (Molar)	Enhancement factor in 446 cm^{-1}	Enhancement factor factor in 1624 cm^{-1}	Limit of detection (Molar)
AgI0 NPs(RLI0)	10^{-6} M<br>10^{-8} M	7.5x10^4<br>1.7x10 ^{5}	1.5x10^5<br>1.5x10^6	10^{-8} M
AgI1 NPs(RLI1)	10^{-6} M<br>10^{-8} M	5.0x10^4<br>3.7x10^5	7.1x10^4<br>5.2x10^5	10^{-8} M
AgI2 NPs(RLI2)	10^{-6} M<br>10^{-8} M	9.2x10^4<br>6.3x10^5	1.0x10^5<br>1.0x10^6	10^{-8} M
AgI3 NPs(RLI3)	10^{-6} M<br>10^{-8}M	1.3x10^5<br>4.3x10^5	1.6x10^5<br>5.4x10^5	10^{-8} M
AgI0 NPs(RFI0)	10^{-6} M<br>10^{-8} M	1.7x10^5<br>7.5x10 ^{5}	2.1x10^5<br>1.0x10^6	10^{-8} M
AgI1 NPs(RFI1)	10^{-6} M<br>10^{-8} M	1.0x10^5<br>6.0x10^5	1.7x10^5<br>1.0x10^6	10^{-8} M
AgI2 NPs(RFI2)	10^{-6} M<br>10^{-8} M	1.3x10^5<br>1.5x10^5	1.3x10^5<br>4.1x10^5	10^{-8} M
AgI3 NPs(RFI3)	10^{-6} M<br>10^{-8} M	1.6x10^5<br>1.6x10^6	1.8x10^5<br>2.9x10^6	10^{-8} M
